# Pinpointing the Onset of Water Harvesting in Reticular Frameworks from Structure

**DOI:** 10.1021/acscentsci.4c01878

**Published:** 2025-02-17

**Authors:** Ha L. Nguyen, Andrea Darù, Saumil Chheda, Ali H. Alawadhi, S. Ephraim Neumann, Lifen Wang, Xuedong Bai, Majed O. Alawad, Christian Borgs, Jennifer T. Chayes, Joachim Sauer, Laura Gagliardi, Omar M. Yaghi

**Affiliations:** † Department of Chemistry, 193248 University of California, Berkeley, California 94720, United States; ‡ Kavli Energy Nanoscience Institute, University of California, Berkeley, California 94720, United States; § Bakar Institute of Digital Materials for the Planet, Division of Computing, Data Science, and Society, University of California, Berkeley, California 94720, United States; ∥ Department of Chemistry, Pritzker School of Molecular Engineering, and Chicago Center for Theoretical Chemistry, 2462 University of Chicago, Chicago, Illinois 60637, United States; ⊥ Beijing National Laboratory for Condensed Matter Physics, Institute of Physics, Chinese Academy of Sciences, Beijing 100190, China; # Songshan Lake Materials Laboratory, Dongguan 530808, China; ∇ School of Physical Sciences, University of Chinese Academy of Sciences, Chinese Academy of Sciences, Beijing 101408, China; ○ KACST−UC Berkeley Center of Excellence for Nanomaterials for Clean Energy Applications, 83527 King Abdulaziz City for Science and Technology, Riyadh 11442, Saudi Arabia; @ Department of Electrical Engineering and Computer Sciences, University of California, Berkeley, California 94720, United States; $ Department of Mathematics, University of California, Berkeley, California 94720, United States; % Department of Statistics, University of California, Berkeley, California 94720, United States; ^ School of Information, University of California, Berkeley, California 94720, United States; & Institut für Chemie, Humboldt-Universität zu Berlin, Berlin 10099, Germany

## Abstract

Covalent organic frameworks (COFs) have emerged as promising atmospheric water harvesters, offering a potential solution to the pressing global issue of water scarcity, which threatens millions of lives worldwide. This study presents a series of 2D COFs, including HCOF-3, HCOF-2, and a newly developed structure named COF-309, designed for optimized water harvesting performance with a high working capacity at low relative humidity. To elucidate their water sorption behavior, we introduce a hydrophilicity index directly linked to intrinsic properties, such as the strength and spatial density of adsorptive sites. This index is mathematically correlated to the step of water adsorption isotherms. Our correlation provides a predictive tool that extends to other microporous COFs and metal–organic frameworks, significantly enhancing the ability to predict their onset positions of water adsorption isotherms based on structural characteristics. This advancement holds the potential to guide the development of more efficient materials for atmospheric water harvesting.

## Graphical abstract

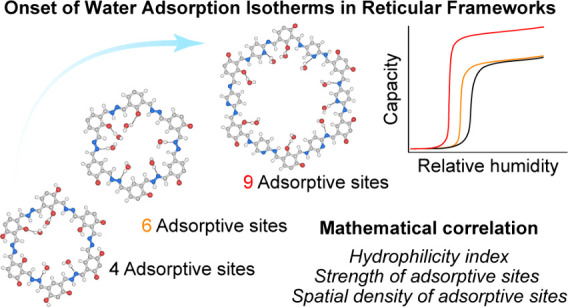

## Introduction

Metal–organic frameworks (MOFs) and covalent organic frameworks (COFs) have emerged as promising materials for water harvesting from air, providing the potential to alleviate the global water crisis.^1–13^ A practical water harvesting material must exhibit at least two important water uptake properties. First, a steep water uptake behavior so that minimal variation in pressure or temperature would be required to collect the harvested water.^7,14^ Second, an onset of water uptake—a position corresponding to approximately 50% of water sorption capacity—at a relative humidity (RH) matching the prevailing humidity level in the region of interest.^15^ While high crystallinity directly governs the steepness of water uptake, predicting the relative humidity at which the onset occurs is heretofore underdeveloped. Herein, we use COFs, which exhibit a highly tunable structure, high crystallinity, chemical stability, and versatile functionalization, as exemplars for how to determine *a priori* the onset of water harvesting from a given porous framework structure. Specifically, we find, contrary to the prevailing understanding, that the density of adsorptive sites (the number of adsorptive sites per unit area or volume) is a vital component in determining the onset position. We derive a mathematical relationship between the onset and the density of adsorptive sites as well as their strength, serving as a predictive descriptor of the water onset position in new microporous COFs and MOFs. Consistent with our newly developed equation, we report a novel water-harvesting COF that demonstrates an exceptional water uptake capacity and a low onset position.

## Results and Discussion

### COF Synthesis, Characterization, and Their Water Sorption

Among the COFs investigated for water harvesting, 2D honeycomb (**hcb**) COFs with hydrazine linkages, such as AB-COF and COF-480-hydrazide,^6,7^ exhibit promising water sorption behavior. However, their imine-bonded structures are prone to hydrolysis. In our efforts to make COFs that take up water at low relative humidity, combined with good water sorption stability upon cycling, we investigated three isoreticular **hcb** frameworks: the previously reported HCOF-3,^16^ HCOF-2,^16^ and a new COF which we named COF-309 (Figure 1).

**Figure 1:**
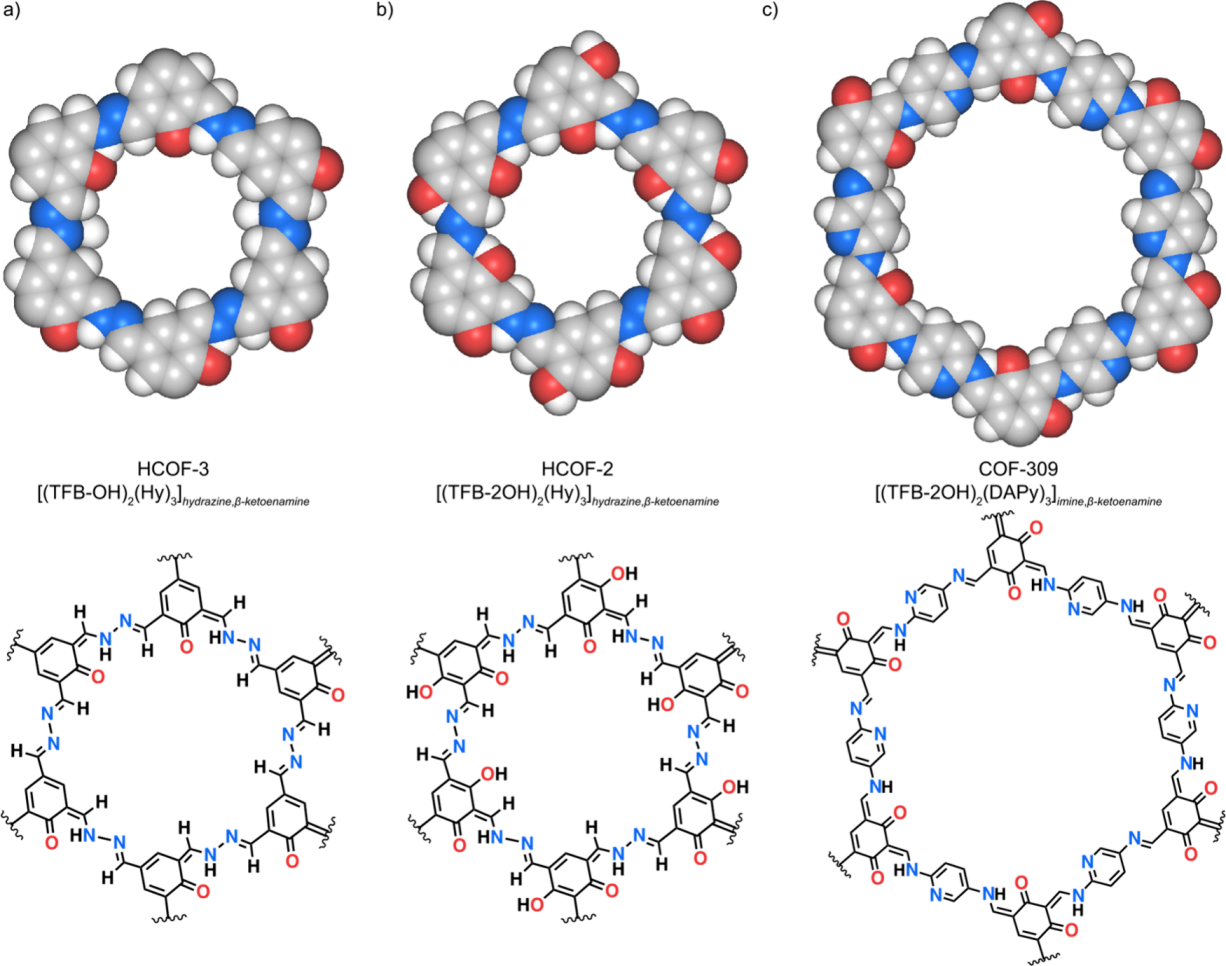
Space filling models and chemical structures corresponding to the single pore fragments of the isoreticular COFs in this study. (a, b) HCOF-3 {[(TFB-OH)_2_(Hy)_3_]_*hydrazine,β‑ketoenamine*_; TFB-OH = 2-hydroxy-1,3,5-triformylbenzene, Hy = hydrazine} and HCOF-2 {[(TFB-2OH)_2_(Hy)_3_]_*hydrazine,β‑ketoenamine*_; TFB-2OH = 2,4-dihydroxy-1,3,5-triformylbenzene} each comprised of hydrazine and β-ketoenamine linkages. (c) COF-309 {[(TFB-2OH)_2_(DAPy)_3_]_*imine,β‑ketoenamine*_; DAPy = 2,5-diaminopyridine} composed of imine and β-ketoenamine linkages. Space filling model color code: C, gray; O, red; N, blue; H, light gray.

HCOF-3 and HCOF-2 were synthesized using modified literature procedures.^16^ COF-309 was prepared by mixing solids of 2,4-dihydroxy-1,3,5-triformylbenzene (TFB-2OH) and 2,5-diaminopyridine (DAPy) in a solution of dioxane, 1,2,4-trichlorobenzene, and aqueous 6 M acetic acid. While these solvents were chosen to ensure optimal solubility and reaction conditions in our proof-of-concept study, we think that exploring greener alternatives (ethanol, water, or other environmentally benign media) is crucial for scaling up. We obtained COF-309 in 96% yield within 3 h by using a microwave-assisted synthesis at 140 °C [Supporting Information (SI) Note 1]. This method indicates a potential for future scalability of COF-309 production.^17,18^ The three COFs were characterized by Fourier-transform infrared spectroscopy (FT-IR), cross-polarization magic angle spinning ^13^C nuclear magnetic resonance (^13^C CP-MAS NMR), elemental analysis (EA), scanning electron microscopy (SEM), thermal gravimetric analysis (TGA), powder X-ray diffraction (PXRD), transmission electron microscopy (TEM), structural optimization using density functional theory (DFT), N_2_ sorption, and H_2_O sorption (SI Notes 1–3, Table S1, and Figures S1–S27).

In evaluating the water uptake behavior of these COFs, we made an unexpected observation. The ratios of hydrazine (which contains 2 adjacent imine functional groups) and β-ketoenamine moieties in HCOF-3 and HCOF-2 are 2:1 and 1:2, respectively. These ratios can only be verified using single-crystal X-ray analysis. Unfortunately, HCOF-2 and HCOF-3 did not crystallize as single crystals, so these ratios remain theoretical. Based on a higher number of more hydrophilic β-ketoenamines in HCOF-2, we anticipated its water onset position to shift to a lower relative humidity than that of HCOF-3. However, the water onset position in HCOF-2 was observed to shift to 32% RH from 23% RH in HCOF-3 (Figure 2).

**Figure 2:**
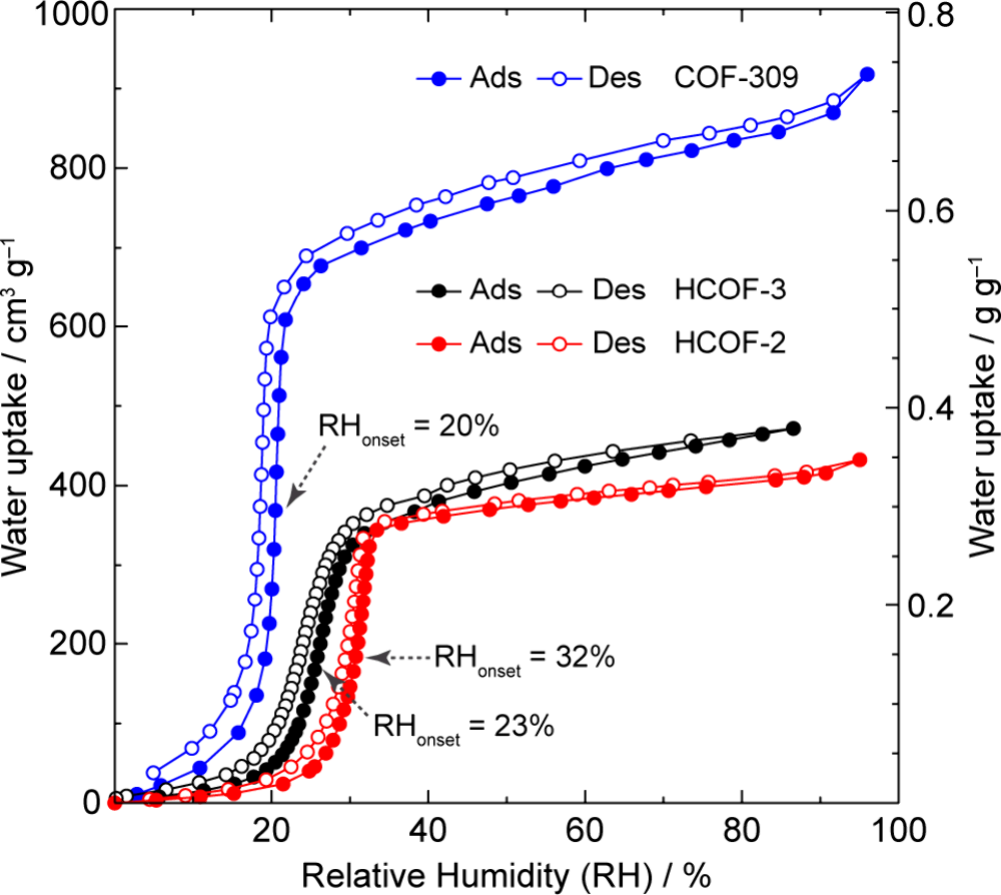
H_2_O sorption measurements of HCOF-2, HCOF-3, and COF-309 at room temperature with onset positions at 32% RH, 23% RH, and 20% RH, respectively. COF-309 exhibits a total water uptake capacity of 0.74 g_water_ g_COF_^–1^.

To gain insight into the chemical reasons for this unexpected behavior, we performed DFT calculations with periodic boundary conditions on the COF unit cell (SI Note 3 and Note 4). We computed the strength of the binding of water to each functional group in the COFs and identified the number of potential adsorptive sites in each pore. The functional groups, ranked by their water binding strength (DFT calculations with the SCAN-D3BJ functional) from highest to lowest, are carbonyl (Δ*E*_Carbonyl–w_ = −16 kcal mol^–1^) greater than imine (Δ*E*_Imine–w_ = −10 kcal mol^–1^) greater than hydroxyl (Δ*E*_Hydroxyl–w_ = −6 kcal mol^–1^). To assess their nucleation ability, we compared these calculated water binding energies to those of two water molecules placed at the center of the COF pore, not interacting with the framework (Δ*E*_w–w_ = −9.4 kcal mol^–1^). We propose that functional groups should yield a water binding energy higher than Δ*E*_w–w_ to be considered as adsorptive sites. These calculations suggested HCOF-3 to have 6 adsorptive sites (2 carbonyl and 4 imine) but HCOF-2 to have only 4 adsorptive sites (2 carbonyl and 2 imine) in each pore fragment (Figure 3). The lower number of adsorptive sites found in HCOF-2 is due to the tautomerization of half of the β-ketoenamine functional groups, as computed using DFT (SI Note 3). The degrees of tautomerism in HCOF-3 and HCOF-2 are 100% and 50%, respectively, based on DFT simulations. The hydroxyl groups were not found to be potential adsorptive sites because their water binding was weakened by hydrogen bond interactions with adjacent imine groups (SI Note 4 and Figures S28–S32). This results in the fact that the hydroxyl groups in HCOF-2 do not adsorb water molecules. The lower number of adsorptive sites in HCOF-2 thus leads to its shift to a higher water onset position.

**Figure 3:**
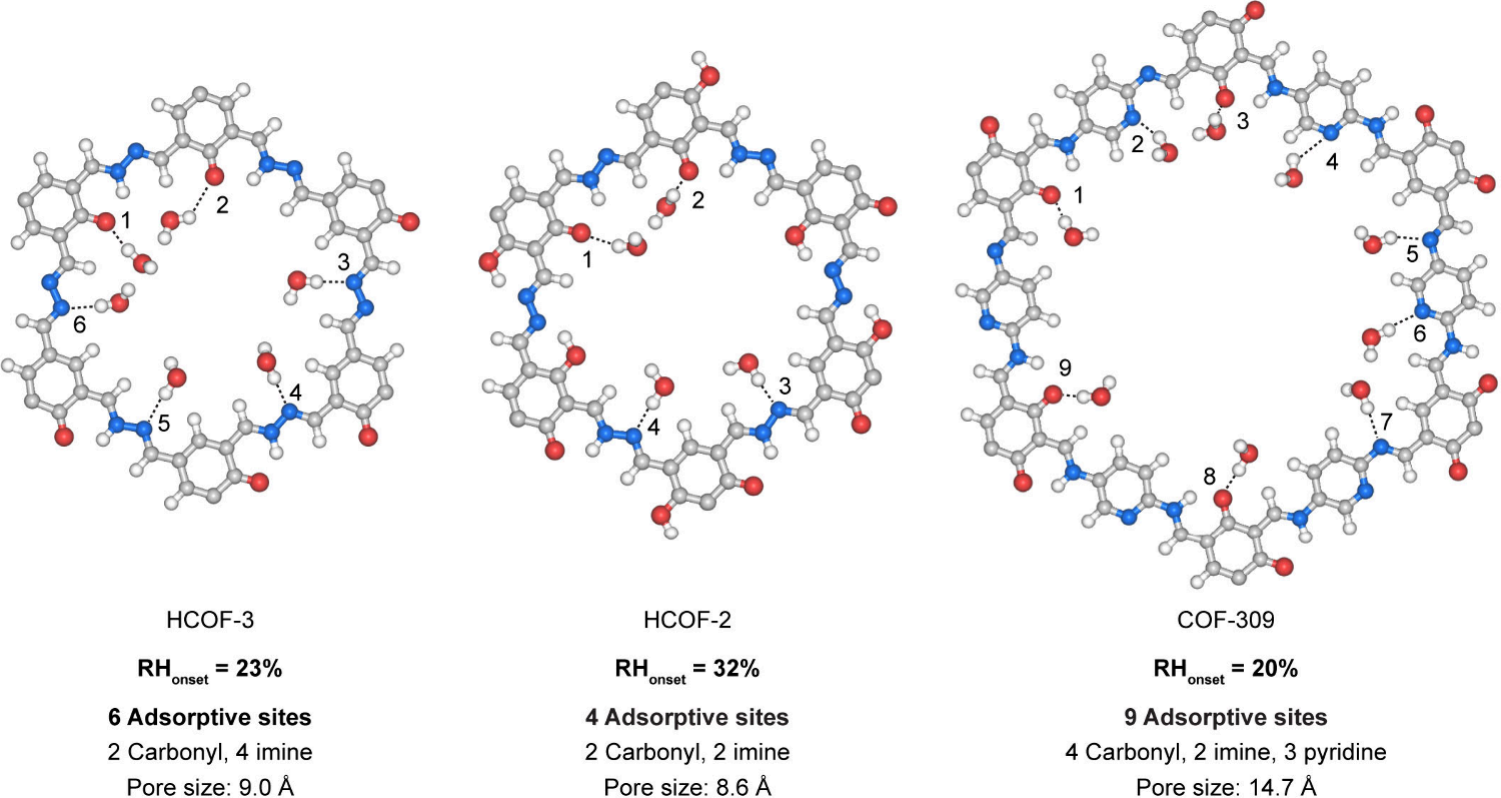
Computed water adsorptive sites in HCOF-3, HCOF-2, and COF-309 exhibiting 6, 4, and 9 water adsorptive sites in each pore fragment, respectively. Color code: C, gray; O, red; N, blue; H, light gray.

Given these findings, we hypothesized that increasing the number of adsorptive sites with water binding energies larger than Δ*E*_w–w_ will shift the onset position of the water adsorption isotherm to a lower relative humidity. Therefore, we designed COF-309, which features 9 adsorptive sites in each pore fragment (4 carbonyl, 2 imine, and 3 pyridine; Δ*E*_Pyridine–w_ = −10 kcal mol^–1^; Figure 3).

COF-309 exhibited a Brunauer–Emmett–Teller surface area of 1698 m^2^ g^–1^ with a pore size distribution at 15.3 Å (Figures S20 and S21) and exceptional water stability (Figures S21 and S22). Its water isotherm, measured at 25 °C, showed a steep step with the onset position at 20% RH and a total water uptake capacity of 0.74 g_water_ g_COF_^–1^ (Figure 2). Additionally, water isotherms measured at 35 and 45 °C showed a negligible loss of the total water uptake capacity (Figure S23). The isosteric heat of adsorption ( $\overset{®}{\Delta H_{\mathrm{ads}}}$ ) of COF-309 was calculated using the Clausius–Clapeyron equation which gave an average value of 48.1 kJ mol^–1^ (11.47 kcal mol^–1^, Figure S24). Gibbs ensemble Monte Carlo simulations further corroborated the water sorption behavior observed in these COFs (SI Note 4 and Figures S26 and S27).

We performed cycling experiments to evaluate the long-term utilization of COF-309 under simulated application conditions. The water isobaric desorption of COF-309 was measured at a water vapor pressure of 1.70 kPa (corresponding to 40% RH at 30 °C) to probe the suitable regeneration temperature (Figure S33), allowing us to determine its desorption temperature of 60 °C. The adsorption–desorption experiments were then conducted under isobaric conditions with a temperature swing between 30 and 60 °C to trigger the release of 0.52 g_water_ g_COF_^–1^ (Figure S34). This value is the highest water working capacity among reported water harvesting COFs. It also surpasses that of established water harvesting MOFs such as CAU-10,^19^ MIL-160,^20^ Al-fumarate,^21^ MOF-303,^22^ MOF-LA2-1­(furan),^23^ and MOF-LA2-2­(furan)^23^ and is comparable to MOF-LA2-1­(pyrazole).^24^ More importantly, the water working capacity of COF-309 remained almost unchanged after at least 170 water adsorption–desorption cycles (Figure S34). It should be noted that, while COF-309 and HCOF-2 are stable under water adsorption–desorption experiments, HCOF-3 is unstable under similar conditions (Figure S7).

### Hydrophilicity Index

The water adsorption isotherms of the COFs studied in this work prompted a detailed examination of the framework properties governing the onset position in water harvesting COFs and MOFs. Particularly, a smaller shift in the onset position was observed between COF-309 (9 adsorptive sites, RH_onset_ = 20%) and HCOF-3 (6 adsorptive sites, RH_onset_ = 23%) in contrast to that between HCOF-3 and HCOF-2 (4 adsorptive sites, RH_onset_ = 32%) despite a larger difference in the number of adsorptive sites between COF-309 and HCOF-3.

Generally, water adsorption in COFs and MOFs occurs in two stages: the first “seeding” stage, characterized by adsorption of primary water molecules onto the framework adsorptive sites, followed by the “pore-filling” stage where secondary water molecules form intermolecular hydrogen-bonded networks with the primary water molecules.^1,2,10,14,19^ We hypothesized that a high density of adsorptive sites within sufficient proximity would favor hydrogen bonding of the secondary water molecules, facilitating the formation of continuous networks between isolated water clusters formed at the adsorptive sites.

We sought to establish a correlation between the onset position of the water adsorption isotherms of microporous COFs and MOFs and the strength and spatial density of their adsorptive sites. We defined the “hydrophilicity index” (*i*_H_; a numerical value that represents a material’s ability to interact with water, considering factors such as the number of adsorptive sites and the strength of their interactions with water) for each framework (eq 1)­

1 $$i_{H}=\frac{N_{\mathrm{ads}}}{S_{A}}\;\exp\left(-\overset{®}{\frac{\Delta H_{\mathrm{ads}}}{RT}}\right)$$

where *N*_ads_ is the number of adsorptive sites per one unit cell (details of *N*_ads_ calculations are provided in SI Note 5); $\overset{®}{\Delta H_{\mathrm{ads}}}$ is the average isosteric heat of water adsorption (kJ mol^–1^), experimentally determined using the Clausius–Clapeyron relationship, which relates to the strength of the adsorptive sites; *R* is the ideal gas constant (J mol^–1^ K^–1^); *T* is the temperature at which the isotherm is measured (K); *S*_A_ is the theoretical surface area per unit cell (m^2^) calculated using N_2_ probe radius (1.82 Å)—theoretical surface area is chosen to minimize deviations in calculations caused by low crystallinity (i.e., low experimental surface area). The hydrophilicity index can be standardized (i.e., made unitless) by multiplying by a factor of 1 × m^2^.

For several microporous water harvesting COFs^4,6,7,10,25^ and MOFs,^14,20,22–24,26,27^ the *i*_H_ of the framework can be used to calculate its onset position of water adsorption (eq 2):

2 $$RH_{onset}={\left(i_{H}\right)}^{-a}={\left(\frac{N_{ads}}{S_{A}}\;\exp\left(-\overset{®}{\frac{\Delta H_{ads}}{RT}}\right)\right)}^{-a}$$

RH_onset_, defined as $\frac{p_{onset}}{p_{\mathrm{sat}}}$ (where *p*_onset_ is the partial pressure of water vapor in kPa and *p*_sat_ is the saturated vapor pressure of water in kPa), represents the onset position of the water sorption isotherm (%). The onset positions of the water adsorption isotherms of MOFs and COFs were found to correlate with their hydrophilicity indices (Figure 4). Detailed methods for determining the hydrophilicity index and number of adsorptive sites are described in the SI (Note 5, Figures S35–S38, and Tables S2 and S3). As an example, we provided step-by-step calculation of the hydrophilicity index for MOF-801.^1^ Additionally, we included an Excel file with calculations of the hydrophilicity indexes for various MOFs and COFs. Among water harvesting COFs, COF-309 stands out as a benchmark structure, exhibiting the lowest onset position at 20% RH. It is noted that we selected framework structures from the literature based on their high performance in atmospheric water harvesting, characterized by high water capacity and low onset of water sorption. Despite their structural diversity, their hydrophilicity indices correlate well with the onset of water harvesting. This demonstrates the broad applicability of our equation in predicting the onset of water harvesting for various framework structures.

**Figure 4:**
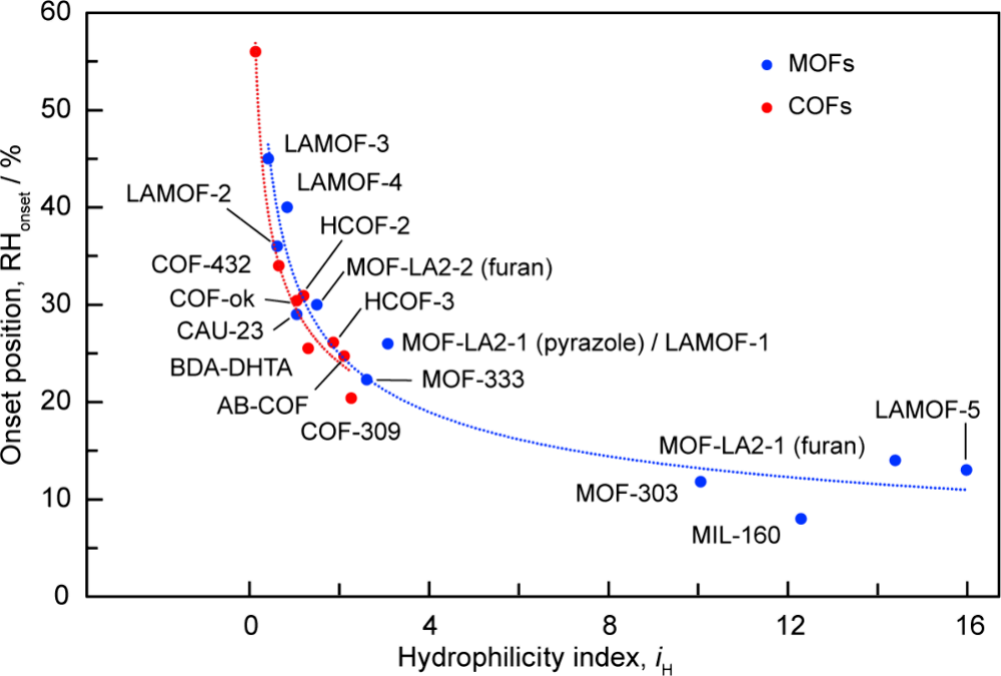
Correlation between the onset position of the water adsorption isotherms of microporous COFs and MOFs to their hydrophilicity index (*i*_H_). As *i*_H_ increases, the onset position of the water adsorption isotherms of the COFs/MOFs reduces, shifting to a lower relative humidity.

Equation 2 can be rearranged to eq 3 to give insights into the thermodynamic origins of the proposed correlation. The nature and number of adsorptive sites effectively govern the free energy for water adsorption in the pores of the COF/MOF. The presence of strong adsorptive sites minimizes the enthalpy of water adsorption, while a higher density of these sites along the pore wall increases the configurational entropy of water molecules adsorbed within the framework. The constant parameter *a* (unitless) is characteristic of the class of microporous structure (COF or MOF) that may account for the long-range interactions of the adsorbed water molecules with the framework.

3 $$RT\;\ln\left(RH_{onset}\right)=RT\;\ln\left(\frac{p_{onset}}{p_{sat}}\right)=a\left(\overset{®}{\Delta H_{ads}}\right)-aRT\;\ln\left(\frac{N_{ads}}{S_{A}}\right)$$

The calculation of *i*_H_ is not limited to the use of experimental $\overset{®}{\Delta H_{\mathrm{ads}}}$ but can also be approximated using estimated electronic energies of hydrogen bonds formed between water molecules and various functional groups (OLSA theory—Omar–Lac Ha–Saumil–Ali theory, SI Note 6 and Table S5). However, *i*_H_ can be more accurately calculated by determining the strength of individual adsorptive sites from more accurate calorimetric measurements or computational studies. It should be noted that we do not intend to use the *i*_H_ equation to describe the complexity of the water adsorption and desorption pathways within the pores of reticular structures. Readers are encouraged to refer to computational simulations reported in the literature.^28,29^

## Conclusion

We report the synthesis, characterization, and water sorption properties of a series of 2D COFs based on imine and β-ketoenamine linkages: HCOF-3, HCOF-2, and COF-309. HCOF-3 and HCOF-2, comprising 6 and 4 adsorptive sites, respectively, exhibit water onset positions at 23% and 32% RH, respectively. In contrast, our newly designed COF-309, having 9 adsorptive sites, displays a step-shaped water sorption isotherm with an onset position at 20% RH. Under isobaric conditions (*p*_water_ = 1.70 kPa) and a temperature swing between 30 and 60 °C, COF-309 exhibits a water working capacity of 0.52 g_water_ g_COF_^–1^ and it is stable for at least 170 water adsorption–desorption cycles. The outstanding water sorption behavior of COF-309 can be attributed to the large pore volume, high spatial density, and strength of the adsorptive sites, which is supported by computational simulations. We derived a correlation between the strength and density of adsorptive sites for microporous reticular frameworks (COFs and MOFs) that successfully predicts the onset position of their respective water adsorption isotherms. Our proposed hydrophilicity index can be used to accelerate the design of new water harvesting frameworks by allowing a quick evaluation of their water onset positions. In conclusion, this work presents a novel, high-performing water harvesting framework (COF-309) with high water uptake capacity and an onset position at low relative humidity. Additionally, we introduce an effective descriptor (hydrophilicity index equation) for predicting the onset positions in water adsorption isotherms. Our equation can be applied to a wide range of microporous framework structures used in atmospheric water harvesting.

## Supplementary Material











## Notes

Synthesis and characterization of COFs including EA, FT-IR spectroscopy, NMR spectra, SEM, TGA, PXRD data, TEM, computational simulations, gas uptake measurements, and water isotherms (PDF)
Crystallographic information file for HCOF-2 (CIF)
Crystallographic information file for HCOF-3 (CIF)
Crystallographic information file for COF-309 (CIF)
Calculations of the hydrophilicity indexes for various MOFs and COFs (XLSX)

## Abbreviations

COFs: covalent organic frameworks
EA: elemental analysis
FT-IR: Fourier-transform infrared spectroscopy
NMR: nuclear magnetic resonance spectroscopy
PXRD: powder X-ray diffraction
TGA: thermogravimetric analysis
SEM: scanning electron microscopy
PXRD: powder X-ray diffraction
TEM: transmission electron microscopy
DFT: density functional theory
