# Supplementary material for: Pinpointing the Onset of Water Harvesting in Reticular Frameworks from Structure
Source: ACS Cent Sci. 2025 Feb 17;11(5):665–71. doi: 10.1021/acscentsci.4c01878 (PMC12123544; doi:10.1021/acscentsci.4c01878)
Supplement: Supplementary file 1 [file oc4c01878_si_001.pdf]

Supporting Information

Pinpointing the Onset of Water Harvesting in Reticular Frameworks from Structure

Ha L. Nguyen[1,2,3], Andrea Darù[4], Saumil Chheda[1,2,3], Ali H. Alawadhi[1,2,3], S. Ephraim Neumann[1,2,3], Lifen Wang[5,6], Xuedong Bai[5,6], Majed O. Alawad[7], Christian Borgs[3,8], Jennifer T. Chayes[3,8,9,10,11], Joachim Sauer[12,*], Laura Gagliardi[4,*], and Omar M. Yaghi[1,2,3,7,*]

[1]Department of Chemistry, University of California, Berkeley, CA 94720, United States

[2]Kavli Energy Nanoscience Institute, University of California, Berkeley, CA 94720, United States

[3]Bakar Institute of Digital Materials for the Planet, Division of Computing, Data Science, and Society, University of California, Berkeley, CA 94720, United States

[4]Department of Chemistry, Pritzker School of Molecular Engineering, and Chicago Center for Theoretical Chemistry, University of Chicago, Chicago, Illinois 60637, United States

[5]Beijing National Laboratory for Condensed Matter Physics, Institute of Physics, Chinese Academy of Sciences, Beijing 100190, China and Songshan Lake Materials Laboratory, Dongguan 530808, China

[6]School of Physical Sciences, University of Chinese Academy of Sciences, Chinese Academy of Sciences, Beijing 101408, China

[7]KACST–UC Berkeley Center of Excellence for Nanomaterials for Clean Energy Applications, King Abdulaziz City for Science and Technology, Riyadh 11442, Saudi Arabia

[8]Department of Electrical Engineering and Computer Sciences, University of California, Berkeley, California 94720, United States

[9]Department of Mathematics, University of California, Berkeley, California 94720, United States

[10]Department of Statistics, University of California, Berkeley, California 94720, United States

[11]School of Information, University of California, Berkeley, California 94720, United States

[12]Institut für Chemie, Humboldt-Universität zu Berlin, Berlin 10099, Germany

*To whom correspondence should be addressed: js@chemie.hu-berlin.de (J.S.), lgagliardi@uchicago.edu (L.G.), yaghi@berkeley.edu (O.M.Y.)

Table of Contents

Supporting Information Note 1 – Chemicals, General Methods, and Synthesis 4

Supporting Information Note 2 – TGA Measurement 13

Supporting Information Note 3 – Computational Modeling 14

Supporting Information Note 4 – Water Sorption Simulations 26

Supporting Information Note 5 – Adsorptive Site Calculation 35

Supporting Information Note 6 – Q_{st} Estimation 42

Supporting Information Note 1 – Chemicals, General Methods, and Synthesis

Chemicals: Benzene-1,3,5-tricarboxaldehyde (TFB), *n*-butanol, 2,5-diaminopyridine (DAPy), dioxane (Dio), hydrazine monohydrate, nitrobenzene, 1,2,4-trichlorobenzene (TCB), and tetrahydrofuran (THF) were purchased from Acros Organic. 1,2-dichlorobenzene (DCB), *N,N′*-dimethylformamide (DMF), glacial acetic acid (AcOH), and mesitylene (Mes) were obtained from Merck Millipore. 1,3,5-Triformylphloroglucinol (Tp) was purchased from Ambeed. 2-Hydroxybenzene-1,3,5-triformylbenzene (TFB-OH) was obtained from ChemScene. 2,4-Dihydroxybenzene-1,3,5- triformylbenzene (TFB-2OH) was synthesized based on literature.[1] Solvents used in this work are not anhydrous; chemicals were used without further purification.

Analytical techniques: Elemental microanalyses (EA) were performed by using a LECO CHNS-932 CHNS elemental analyzer. The COF was synthesized using CEM Discover SP microwave synthesizer (CEM corporation). FT-IR spectra were collected in-house using a Bruker ALPHA Platinum ATR-FT-IR Spectrometer equipped with a single reflection diamond ATR module. Helium gas was employed to correct for dead volume. Scanning electron microscopy (SEM) images were obtained on a Zeiss XB 550 high-resolution SEM with an accelerating voltage of 1.0 kV. The samples were dispersed on conductive carbon tape, mounted on stubs, and sputter coated (Pd/Au) with a Tousimis sputter coater on top of a Bio-Rad E5400 controller. Transmission electron microscopy images were obtained using a Cs-corrected JEOL Grand ARM 300 electron microscope operating at 300 kV, utilized with a K2 camera for low-dose imaging. The specimens were dispersed directly onto ultrathin carbon-coated copper grids and affixed to the grids via electrostatic adsorption. Solution-state NMR spectra were collected using a Bruker NEO 500 spectrometer and solid-state NMR spectra were collected using a 11.4 T magnet on a Bruker Advance I spectrometer operating at 125.75 MHz for ^{13}C-NMR. Thermogravimetric analysis (TGA) curves were recorded on a TA Q500 thermal analysis system under nitrogen flow. Powder X-ray diffraction (PXRD) data was collected on a Bruker D8 Advance diffractometer (Bragg–Brentano geometry; CuKα1, λ = 1.54059 Å). N_2 sorption measurements were carried out on a Micromeritics ASAP 2420 surface area analyzer. A liquid N_2 bath was used for measurements at 77 K. Ultrahigh-purity-grade N_2 and He (99.999% purity) were used throughout sorption experiments. Isothermal water vapor measurements were performed using the Belsorp MAX II high precision gas/vapor adsorption measurement instrument. Prior to measurements, the vapor source containing deionized water (analyte) was degassed using three cycles of freeze-pump-thaw.

To maintain a constant temperature for each respective measurement, sample cells were immersed in an isothermal water bath throughout the measurement. Isobaric cycling water measurements were conducted with the TA Instruments SDT Q600 Thermogravimetric Analyzer & Differential Scanning Calorimeter. A humidified nitrogen gas stream was generated by passing nitrogen gas through a water bubbler system held at a constant temperature. The water vapor partial pressure was maintained by controlling the ratio between dry and humidified nitrogen gas feeds that were mixed then fed into the TGA chamber. The combined flow rate of both gas feeds was maintained at 200 mL min^{-1}. Downstream of the TGA chamber, the gas feed's temperature and humidity were measured using dedicated sensors.

Synthesis of HCOF-2 An 18 × 150 mm borosilicate glass tube (Type I, Class B, Chemglass) was charged with TFB-2OH (20.5 mg, 0.11 mmol), hydrazine monohydrate (24 μL, 0.49 mmol), and 1 mL of nitrobenzene. The solution was sonicated for 2 min before adding mesitylene (0.25 mL) and trifluoroacetic acid (TFA; 8 μL). The tube was purged with nitrogen for 2 min and heated to 120 °C for 72 h yielding an orange solid HCOF-2. The solid was filtered, washed with DMF (5 mL × 3 times), chloroform (5 mL × 3 times), and dried under air. HCOF-2 was solvent exchanged with chloroform in a Soxhlet extractor for 5 h. HCOF-2 was finally activated under dynamic vacuum at room temperature for 0.5 h followed by dynamic vacuum at 120 °C for 5 h (12.2 mg HCOF-2 obtained, 59% yield based on TFB-2OH).

Synthesis of HCOF-3 A Pyrex glass tube measuring 10 × 8 mm (outer diam. × inner diam.) was loaded with TFB-OH (27.5 mg, 0.15 mmol), 0.50 mL of DCB, and 0.05 mL of *n*-butanol. The solution was sonicated for 1 min. Subsequently, 0.45 mL DCB containing 15 μL of hydrazine monohydrate (0.31 mmol) was added into the solution. Aqueous 6M acetic acid (0.34 mL) was then added as a catalyst. The tube was flash frozen at 77 K under liquid N_2, evacuated to an internal pressure of 120 mTorr and flame sealed. The reaction was heated in a preheated oven at 120 °C for 3 days. The crude white solid was cooled down to room temperature, isolated by filtration, and washed with DMF (5 mL × 3 times) and chloroform (5 mL × 3 times). HCOF-3 was then washed with chloroform in a Soxhlet extractor for 5 h and was finally activated under dynamic vacuum at room temperature for 1 h followed by

dynamic vacuum at 120 °C for 5 h (17.4 mg HCOF-3 obtained, 67% yield based on TFB-OH).

Synthesis of COF-309 COF-309 was synthesized using microwave-assisted method. In general, 28 mg TFB-2OH (0.144 mmol) and 31 mg (0.284 mmol) DAPy were loaded into an 8-mL microwave tube before being vortexed for a few sec. A mixture of dioxane and 1,2,4-trichlorobenzene (0.7:0.3 mL) was added into the microwave tube. The solution was vortexed for a few sec and 0.36 mL of aqueous acetic acid 6M was subsequently added to the solution. The microwave tube was again vortexed for a few seconds before being sealed with a silicone cap. The tube was then transferred to a CEM Discover SP microwave synthesizer. The COF was synthesized at 140 °C for 3 hours. The microwave synthesizer was fixed at 300 W power and 250 psi pressure. The combination of dioxane, 1,2-dichlorobenzene, and aqueous 6M acetic acid (0.75:0.25:0.32 mL) at 120 °C and 3 hours also gave rise to COF-309 with similar crystallinity and porosity.

The crude product was filtered out and washed with DMF several times (20 to 30 mL DMF each time) until obtaining the clear solution. COF-309 was solvent exchanged with acetone at least 5 hours using a Soxhlet extractor. Activated COF-309 (43 mg, 96% yield based on TFB-2OH) was obtained by heating the solvent-exchanged sample under dynamic vacuum at 120 °C for 8 hours. Elemental analysis of activated COF-309: Calcd. for $C_{33}H_{21}N_9O_4$: C, 65.23; H, 3.48; N, 20.75%. Found: C, 62.58; H, 3.80; N, 19.87%. The deviation between theoretical %C and experimental %C is due to the presence of one molecule of DMF which cannot be fully removed through solvent exchange and activation steps. The presence of DMF in COF-309 is proven by solid-state ^{13}C-NMR.

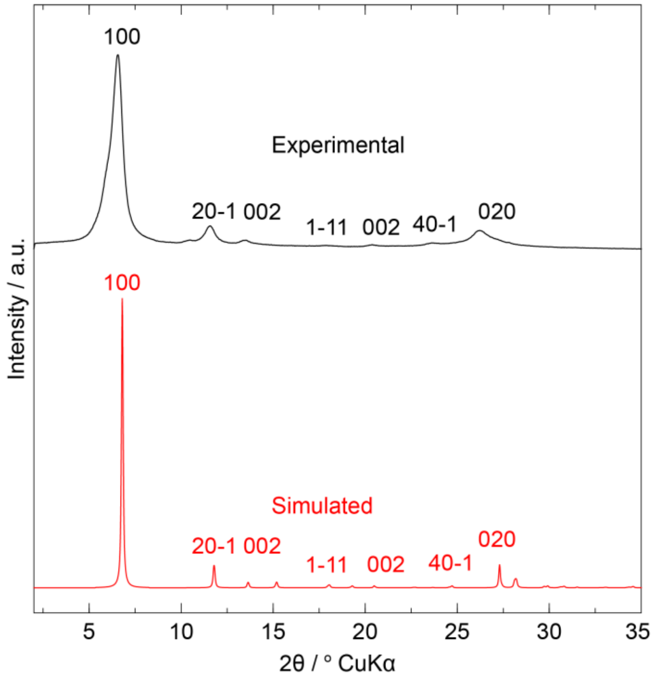

Figure S1. Overlay of the simulated PXRD pattern and experimental pattern of serrated HCOF-2.

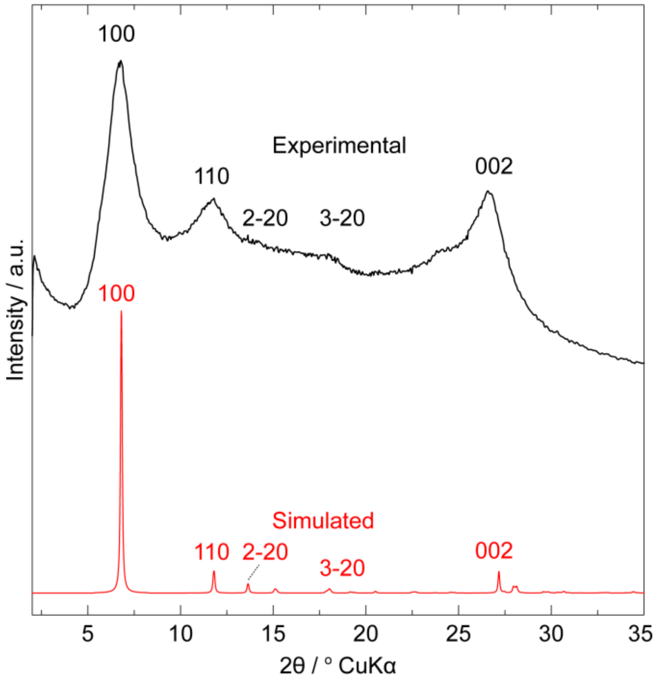

Figure S2. Overlay of the simulated PXRD pattern and experimental pattern of serrated HCOF-3.

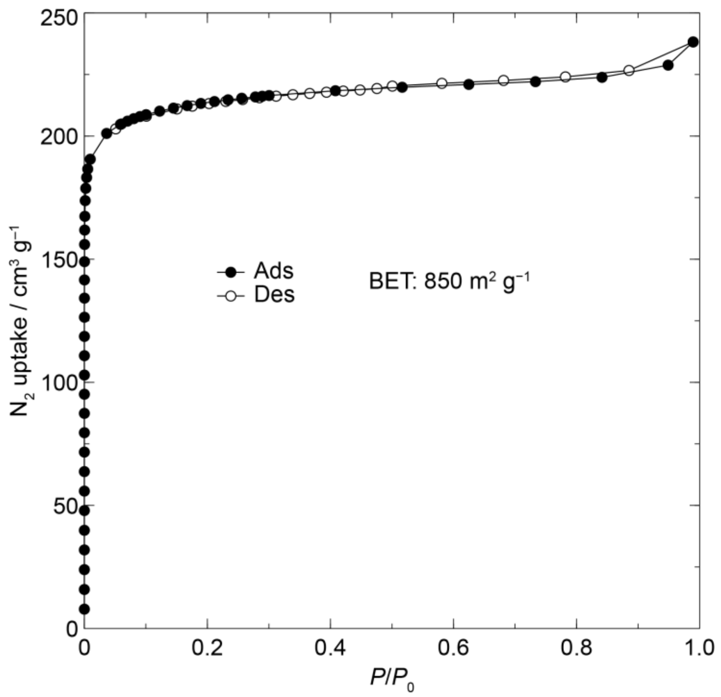

Figure S3. N_2 sorption isotherm at 77 K of activated HCOF-2. The filled and open circles represent the adsorption and desorption branches, respectively.

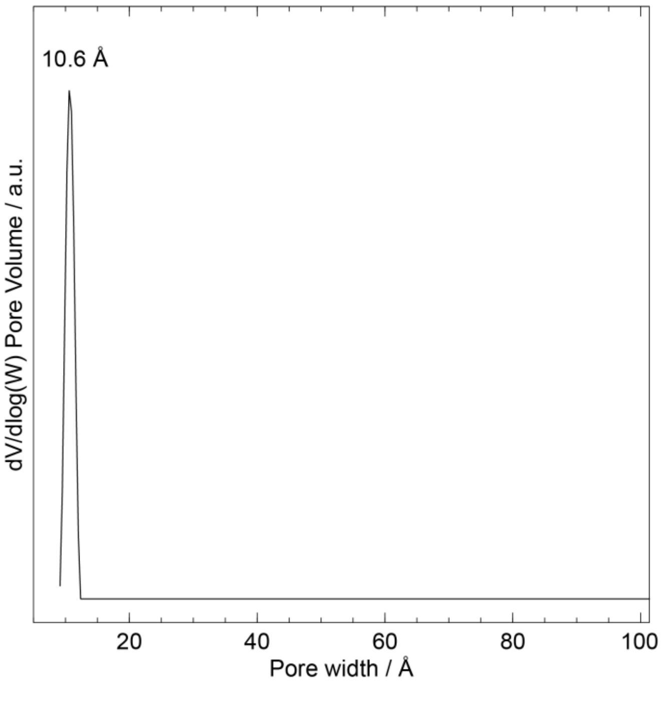

Figure S4. Pore size distributions of HCOF-2. The N_2-Cylindrical Pores-Oxide Surface model was used indicating a pore width of 10.6 Å which is similar to the model structure (10 Å).

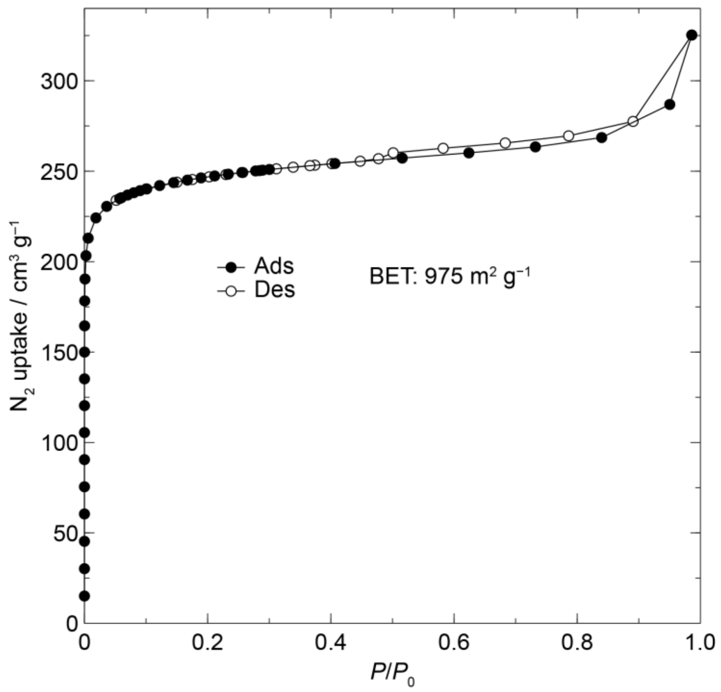

Figure S5. N_2 sorption isotherm at 77 K of activated HCOF-3. The filled and open circles represent the adsorption and desorption branches, respectively.

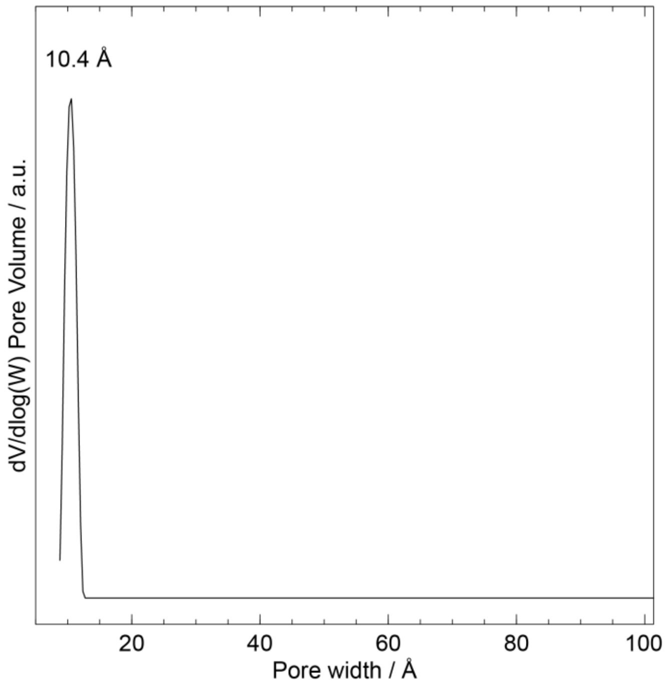

Figure S6. Pore size distributions of HCOF-3. The N_2-Cylindrical Pores-Oxide Surface model was used indicating a pore width of 10.4 Å which is similar to the model structure (11 Å).

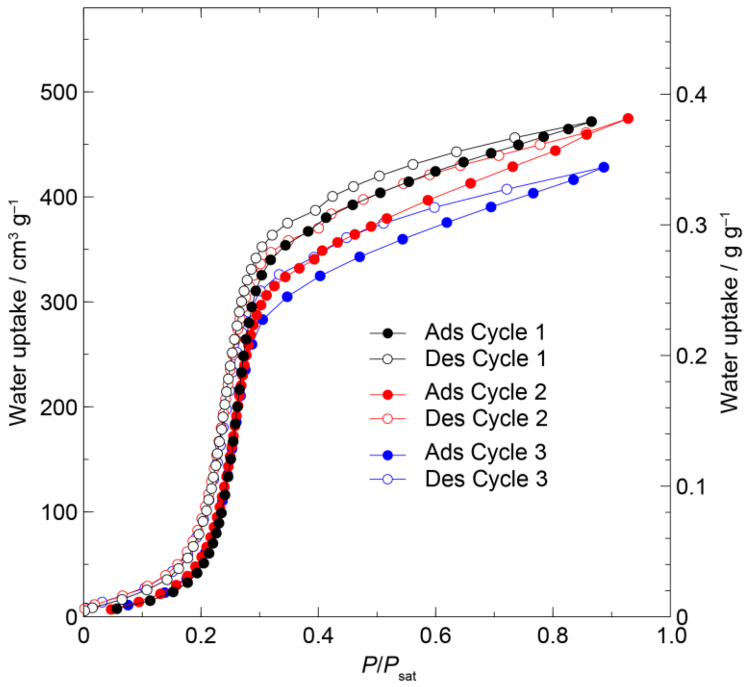

Figure S7. Three cycles of water sorption analysis of HCOF-3 at room temperature. Water capacity reduced after the first cycle, indicating that HCOF-3 is not stable under water sorption conditions. *P*: Water vapor pressure. P_{sat}: The saturation vapor pressure at the respective temperature. The filled and open circles represent the adsorption and desorption points, respectively.

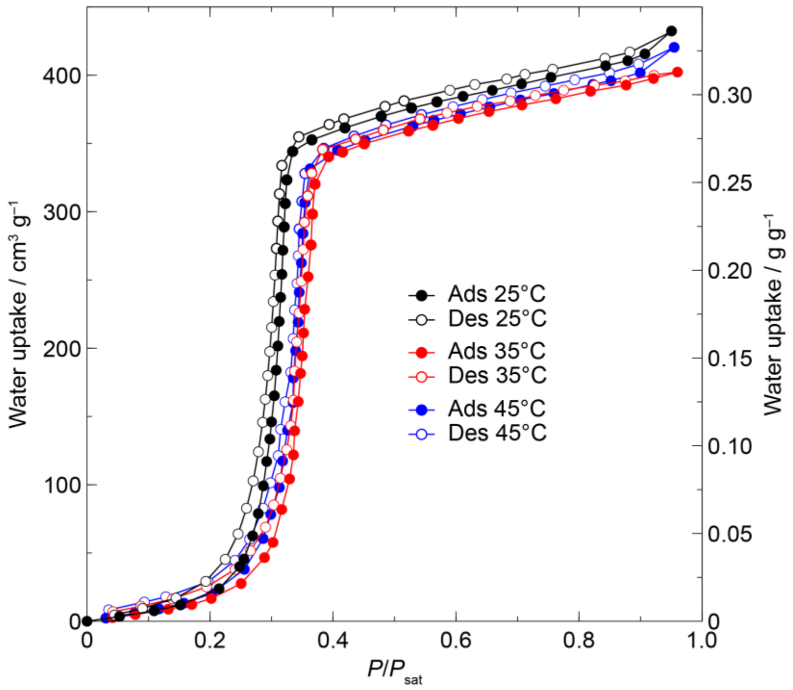

Figure S8. Water sorption analysis of HCOF-2 at different temperatures (25 °C, 35 °C, and 45 °C). *P*: Water vapor pressure. P_{sat}: The saturation vapor pressure at the respective temperature. The filled and open

circles represent the adsorption and desorption points, respectively. P: Water vapor pressure. P_{sat}: The saturation vapor pressure at the respective temperature. The filled and open circles represent the adsorption and desorption points, respectively.

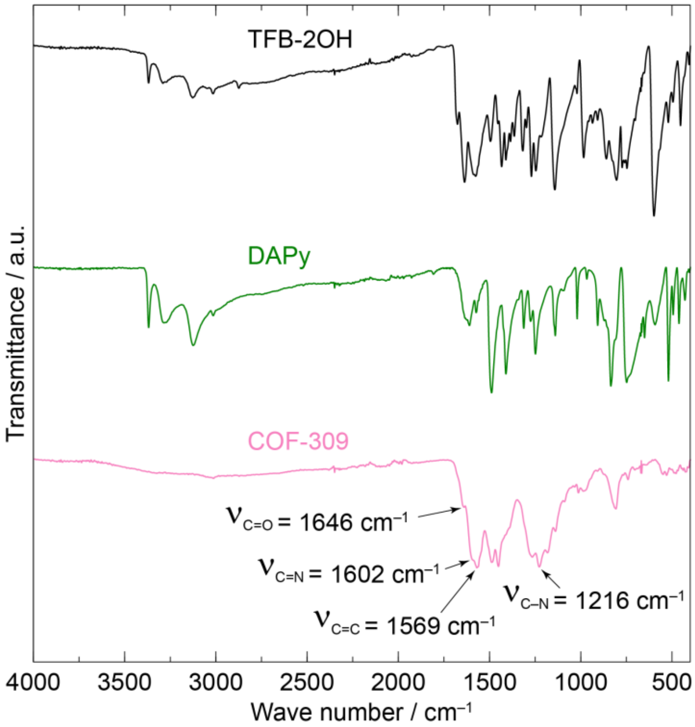

Figure S9. Overlay of the FT-IR spectra of TFB-2OH, DAPy, and COF-309. The new bands at 1646 cm^{-1}, 1602 cm^{-1}, 1569 cm^{-1}, and 1216 cm^{-1} are assigned to C=O, C=N, C=C, and C–N bonds in one imine andtwo β-ketoenamine linkages in the COF-309 framework.

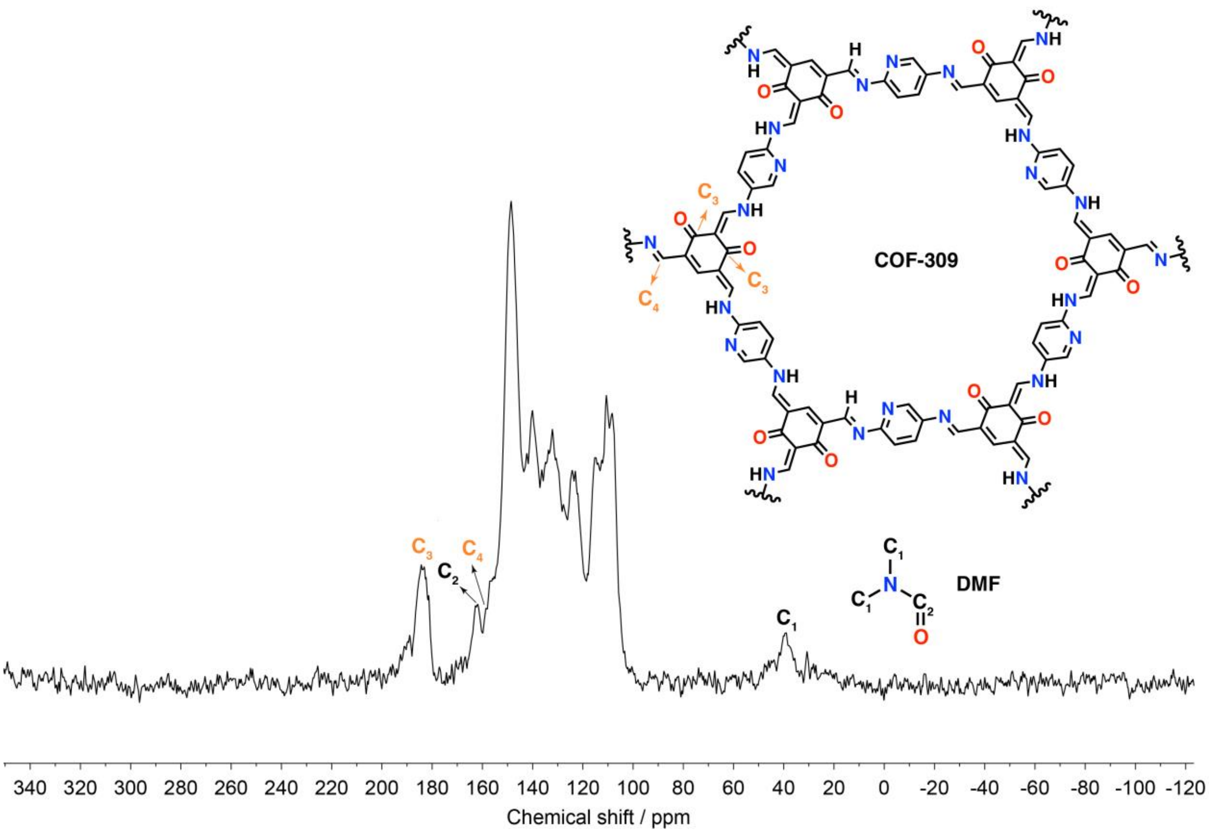

Figure S10. Solid-state ^{13}C-NMR spectroscopy of COF-309 showing characteristic peaks of imine and β-ketoenamine linkages of the framework alongside DMF guest molecule retaining in the COF structure. The peak assignments were validated by comparison with solution-state ^{13}C-NMR of TFB-2OH (Figure S9 in the SI of Ref 2 similar model compound (Figure 3a of Ref 3 and previously reported COF (Figure S8 in the SI of Ref 4).

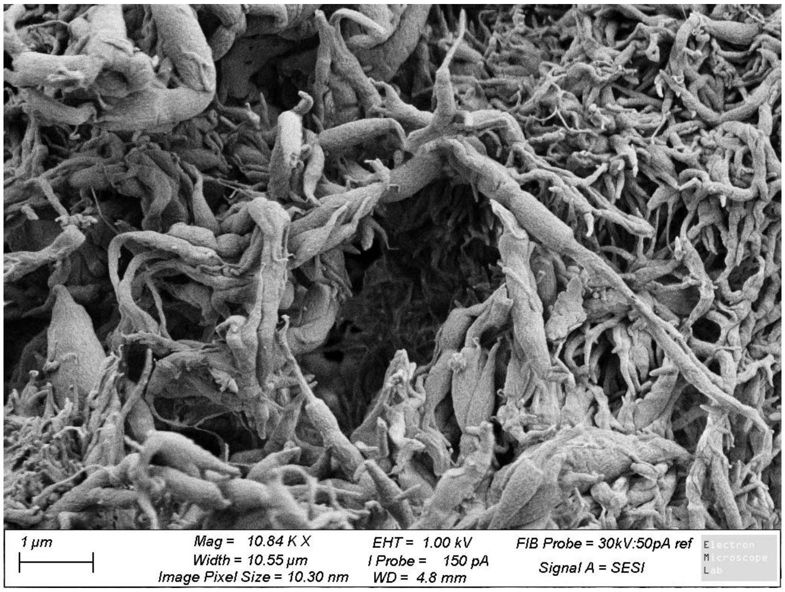

Figure S11. SEM image of COF-309 showing root-shaped crystalline solids at 10.84 kX magnification.

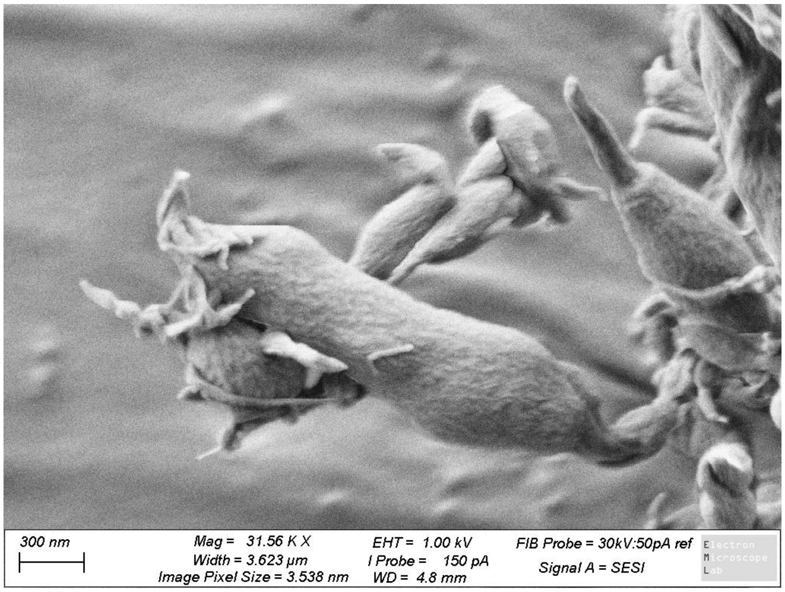

Figure S12. SEM image of COF-309 showing root-shaped crystalline solids at 31.56 kX magnification.

Supporting Information Note 2 – TGA Measurement

The thermal stability of COF-309 was studied by thermogravimetric analysis. The activated COF-309 (ca. 4.0 mg) was heated from 25 to 800 °C with a gradient of 5 °C min^{-1} under air flow (60 mL min^{-1}).

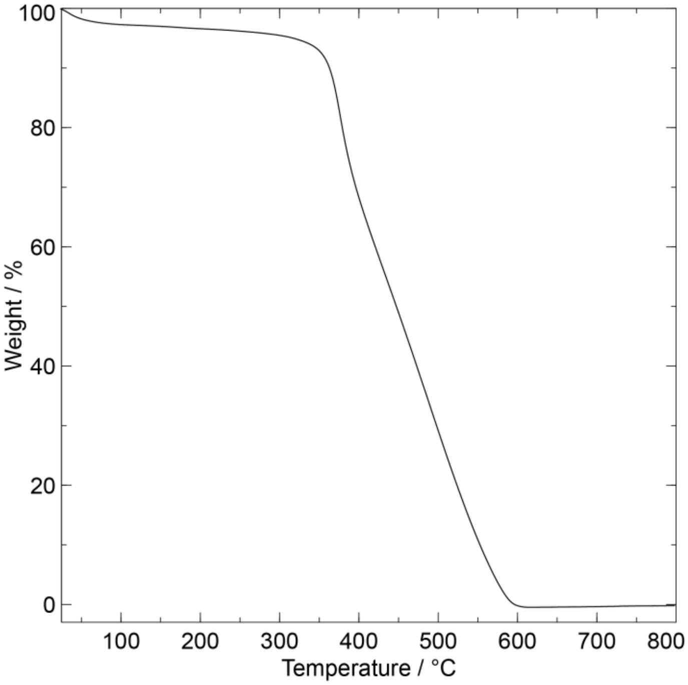

Figure S13. Thermalgravimetric analysis of COF-309 under air flow.

Supporting Information Note 3 – Computational Modeling

Structural model of COF-309

Crystal structure of COF-309 was first simulated using the *Materials Visualizer* module of *Materials Studio* 8.0 software[5]. Upon completion of the structural model, an energetic minimization was performed using the universal force field implemented in the *Forcite* module of *Materials Studio*. During this process, the unit cell parameters were optimized until proper convergence was achieved (energy convergence criteria were set to 10^{-4} kcal mol^{-1}). The stable structure of COF-309 was optimized using density functional theory periodic boundary conditions.

Computational details

Periodic boundary condition density functional theory (PBC-DFT) calculations were performed on a single unit cell of each considered species using the Vienna Ab Initio Simulation Package (VASP 6.2.1)[6]. R^{2}SCAN[7] meta-GGA density functional was used along with Grimme's D3 dispersion correction with Becke-Johnson damping (D3BJ)[8]. A plane-wave basis[9,10] set with a kinetic energy cut-off of 520 eV was used for the geometry optimizations, and normal pseudopotential for all atoms. Energy and force convergence criteria of 10^{-6} eV and 0.01 eV Å^{-1}, respectively, were employed for all optimizations. A 1 × 1 × 2, and a 1 × 1 × 3 Γ-centered k-point grid was used for the Brillouin zone sampling for HCOF-2 and HCOF-3, and COF-309 respectively. Single-point calculations were performed using the functionals SCAN[11], and B3LYP[12–15] including D3BJ dispersion correction. A plane-wave basis set with a kinetic energy cutoff of 910 eV was used and a hard pseudopotential for all atoms, and energy convergence criterion of 10^{-8} eV for SCAN, and 10^{-6} for B3LYP. Vibrational frequency calculation (IBRION = 5) at 298 K was done at the optimization level of theory and used to define the nature of the stationary points involved in the reaction mechanism. The free energy correction (G_{corr}) applied to the electronic energy was calculated based on transaltional, rotational, and vibrational degrees of freedom from such frequency calculations.

Gibbs ensemble Monte Carlo details

NpT-GEMC simulations was performed with the Monte Carlo for Complex Chemical Systems-Minnesota software (MCCCSMN)[16] using a simulated gas-phase reservoir containing water molecule explicitly to account for intermolecular interactions. Saturation pressure (P_{sat}) is obtained through separate GEMC simulations so that adsorption isotherms can be shown as a function of

relative pressure (P/P_{sat}, RH). A 2 × 2 × 5 supercell of every DFT-optimized COF structure was used for the simulations. COFs were optimized with explicit water molecules coordinated to the hydrophilic substituent groups (CO, OH, and N), such water molecules are then removed before creating the supercell, and the COF structure used without any further modification. The COF structure is kept frozen throughout the GEMC simulation. Full details are provided in Supporting Information Note 4.

Structural optimization of COF structures at the DFT level

All the COF structures have been thoroughly optimized considering inter- and intra- molecular conformations. The intermolecular features are related to the layering, indeed these 2D COF structures are formed by single sheets that can stack on top of each other's with different dispositions. These layering types are equatorial, inclined, serrated, and staggered which have been made manually starting from a single COF layer and shifting the subsequent layer to generate such patterns. Multiple shifts have been performed, and in Figure S14 shown as graphical representation. We reported the most stable optimized structures in Table S1a, 1b, and 1c with their relative electronic energy and free energy difference between staking modes. Structural optimization and single point calculations are done as described in the computational details.

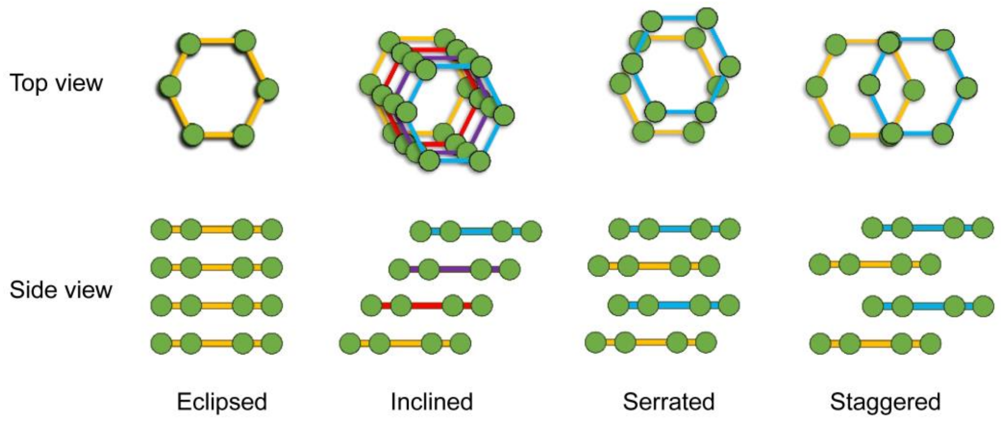

Figure S14. Graphical representation of possible conformational structures of 2D honeycomb stacking mode.

Table S1a. Relative electronic and free energy values in kcal mol^{-1} (T = 298 K) and quasi-harmonic free energy correction in eV, for different COF layering in gas phase of COF-309. Serrated is the most stable layering, thus the relative energetics are related to such structure. R^{2}SCAN (energy cut-off for the plane-wave basis set at 520 eV) has been used for geometry optimization; SCAN and B3LYP for single point calculations (energy cut-off for the plane-wave

basis set at 910 eV). All functionals include D3BJ empirical dispersion. (See computational details section)

Name	ΔE				ΔG		
	R^2SCAN	SCAN	B3LYP	G_{corr}	R^2SCAN	SCAN	B3LYP
**COF-309 eclipsed**	39.3	41.2	40.4	12.6	40.3	42.3	41.4
**COF-309 serrated**	0.0	0.0	0.0	25.1	0.0	0.0	0.0
**COF-309 staggered**	14.0	20.6	20.5	25.2	18.3	24.9	24.8
**COF-309 inclined**	8.3	9.3	8.2	12.7	15.7	16.7	15.6

Table S1b. Relative electronic and free energy values in kcal mol^{-1} (T = 298 K) and quasi-harmonic free energy correction in eV, for different COF layering in gas phase of HCOF-2. Serrated is the most stable layering, thus the relative energetics are related to such structure. R^2SCAN (energy cut-off for the plane-wave basis set at 520 eV) has been used for geometry optimization; SCAN and B3LYP for single point calculations (energy cut-off for the plane-wave basis set at 910 eV). All functionals include D3BJ empirical dispersion. (See computational details section)

Name	ΔE				ΔG		
	R^2SCAN	SCAN	B3LYP	G_{corr}	R^2SCAN	SCAN	B3LYP
**HCOF-2 eclipsed**	72.1	70.1	67.4	7.1	63.4	61.5	38.7
**HCOF-2 (2 C=O, 2 C-OH) serrated**	2.4	–0.35	1.26	7.3	2.18	–0.6	1.1
**HCOF-2 (all C-OH) serrated**	0.0	0.0	0.0	14.6	0.0	0.0	0.0
**HCOF-2 inclined**	4.9	0.8	0.6	7.3	4.9	0.8	0.6

Table S1c. Relative electronic and free energy values in kcal mol^{-1} (T = 298 K) and quasi-harmonic free energy correction in eV, for different COF layering in gas phase of HCOF-3. Serrated is the most stable layering, thus the relative energetics are related to such structure.

R^2SCAN (energy cut-off for the plane-wave basis set at 520 eV) has been used for geometry optimization; SCAN and B3LYP for single point calculations (energy cut-off for the plane-wave basis set at 910 eV). All functionals include D3BJ empirical dispersion. (See computational details section)

**Name**	**Δ*E***				**Δ*G***		
	R^2SCAN	SCAN	B3LYP	G_{corr}	R^2SCAN	SCAN	B3LYP
**HCOF-3 eclipsed**	15.5	19.2	17.7	7.1	15.5	19.1	17.7
**HCOF-3 (all C=O) serrated**	8.1	10.8	13.8	14.1	6.0	8.8	11.7
**HCOF-3 (all C-OH) serrated**	0.0	0.0	0.0	14.2	0.0	0.0	0.0
**HCOF-3 inclined**	66.6	36.7	38.2	7.3	75.4	45.5	47.0

Following is the description of every COF structure optimization. We considered the pristine structure (*dry*) and a *wet* structure, where explicit molecules of water are added into the pore of every COF structure to simulate the adsorption process and better describe the structural deformation caused by the presence of water in the pores.

Starting with HCOF-3, the most stable layering turned out to be the serrated one. The pristine structure shows the presence of enol-imine functional groups in the pore (Figure S15). On the other hand, when explicit water molecules are added to the pore, in correspondence to every hydrophilic group present in the pore (OH, and imine N) the tautomerization process takes place isomerizing every enol-imine groups (OH and N imine) into keto-enamine groups (carbonyl and NH amine) (Figure S15). To computationally address the tautomerization in HCOF-3 we optimized the HCOF-3 structure in gas phase without presence of explicit water molecules (*dry* condition) and in the presence of six water molecules per layer (thus 12 per unit cell, *wet* condition) each of them bound to the hydrophilic group of the pore. The free energy associated to the tautomerization heading to the keto-enamine groups in dry condition, is endergonic of 8.8 kcal mol^{-1} calculated with SCAN-D3BJ functional. Due to the presence of four enol-imine functional groups, this value has to be divided by four, giving 2.2 kcal mol^{-1} per every enol-imine isomerizing into keto-enol

groups, thus confirming the most stable enol-imine COF isomer in the crystal structure (Table S1). On the other hand, in *wet* conditions, this value becomes 0.7 kcal mol^{-1} suggesting an equilibrium between the two tautomers facilitated by the presence of water. The keto-enol tautomer optimized in wet conditions will be subsequently use for further calculations such as the isotherm prediction with Monte Carlo simulations, after removal of such water molecules.

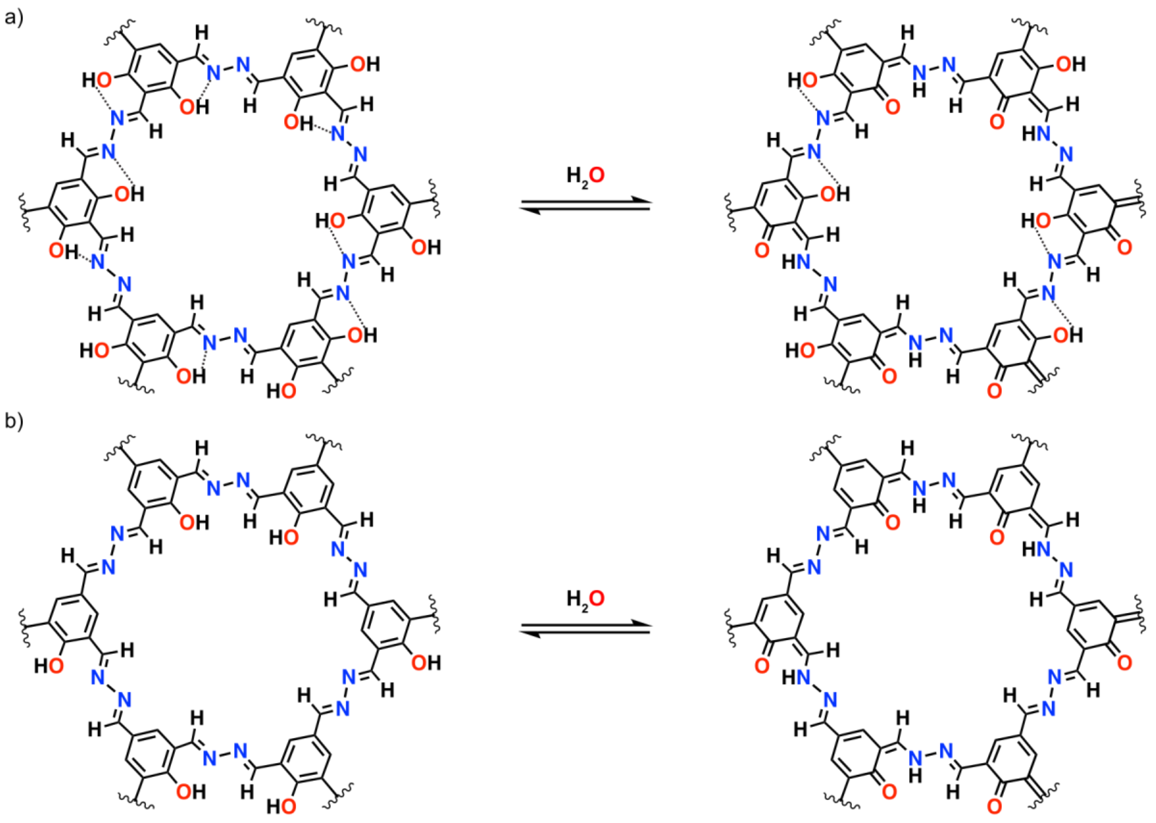

Figure S15. Graphical representation of the equilibrium tautomerization of HCOF-2 (a) and HCOF-3 (b).

The most stable layering of HCOF-2 is a mix of serrated and inclined, the free energy difference between these staking modes is about 1 kcal mol^{-1} at both SCAN and B3LYP functionals (Table S1). This small free energy difference could be the explanation for the very low crystallinity observed experimentally for such structure, suggesting the presence of these two different layering in the experimental crystal structure. As for HCOF-3, the pristine (*dry*) crystal structure of HCOF-2 presents all enol-imine functinoal groups; however, in the presence of explicit water molecules the tautomerization can take place (as observed for HCOF-3), transforming the enol-imine groups (OH and N imine) into keto-enamine groups (carbonyl and NH amine), although, differently that in HCOF-3, the tautomerization of HCOF-2 is partial. Indeed, after structural optimization in the

presence of water molecules (with R^2SCAN functional), among four enol-imine groups only two of them are isomerized to the keto-enamine ones (Figure S15). The isomers with all keto-enamine, or all enol-imine were not found after several trial of structural optimization of such initial structures. Similarly to HCOF-3, six water molecules were manually placed per layer (thus 12 per unit cell) of HCOF-2 in correspondence to the hydroxyl and imine groups. The optimized structure of HCOF-2 with explicit water molecules is then used to perform the isotherm simulation by previous removal of such water molecules.

The COF-309 initial structure was taken from the experimental PXRD and optimized at the DFT level using R^2SCAN functional in gas phase. Geometrically, the serrated layering is the most stable without any other stacking mode close in electronic energy terms. As done for previous COF structures, explicit water molecules are added to fill the COF pore, followed by geometry optimization. No tautomerization has been found in this case, indeed the energy difference from the COF-309 serrated structure carrying enol-imine groups is 22.4 kcal/mol higher compared to the one carrying keto-enol groups. These structural features are confirming the high stability of COF-309, compared to HCOF-2 and HCOF-3 that are affected by equilibrium tautomerization and isoenergetic stacking modes.

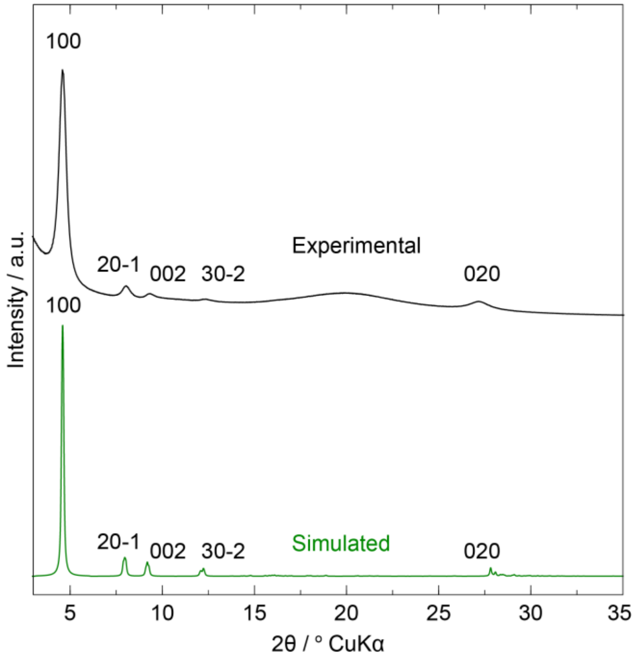

Figure S16. Overlay of the simulated PXRD pattern and experimental pattern of serrated COF-309. The 020 peak in the experimental PXRD pattern shifts to the lower 2θ due possibly to the presence of DMF intercalating between the COF layers which is supported by ^{13}C CP-MAS NMR (Figure S10).

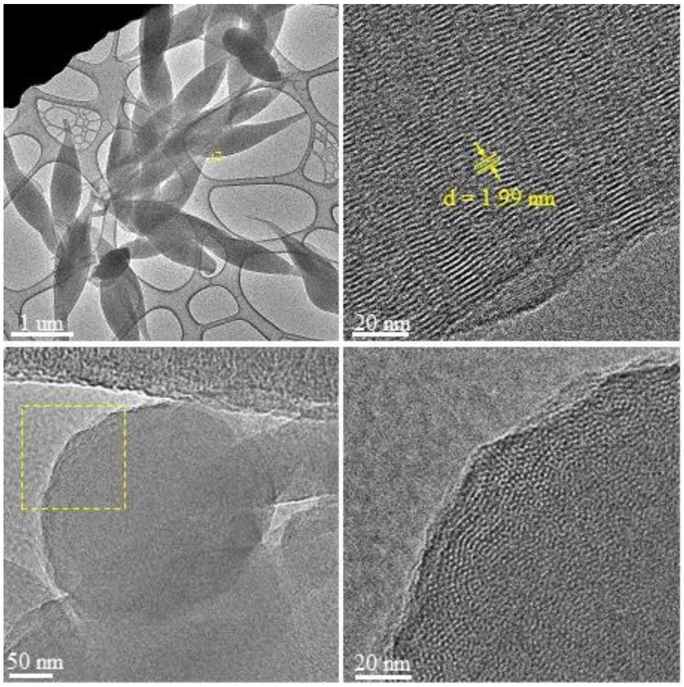

Figure S17. TEM images of activated COF-309 at different scales. The sample exhibits root-shaped morphology in consistent with SEM results. The zoom-in TEM image (at 20-nm scale) shows d-Spacing of 1.99 nm (19.9 Å) corresponding to the 100 lattice planes in consistent with the first line in the simulated PXRD pattern. Owning to its beam-sensitive nature, the specimen experiences amorphization during imaging, posing a challenge to subsequent high-resolution characterization.

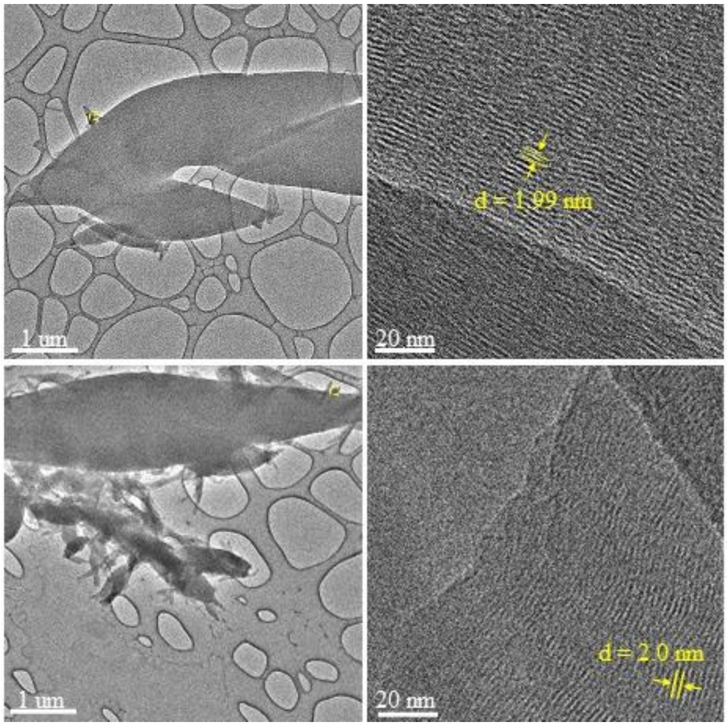

Figure S18. TEM images at different scales of activated COF-309 exposed under 95% RH at room temperature overnight. d-Spacing of ~2.0 nm (20 Å) corresponds to the 100 lattice planes (the first line in the simulated PXRD pattern).

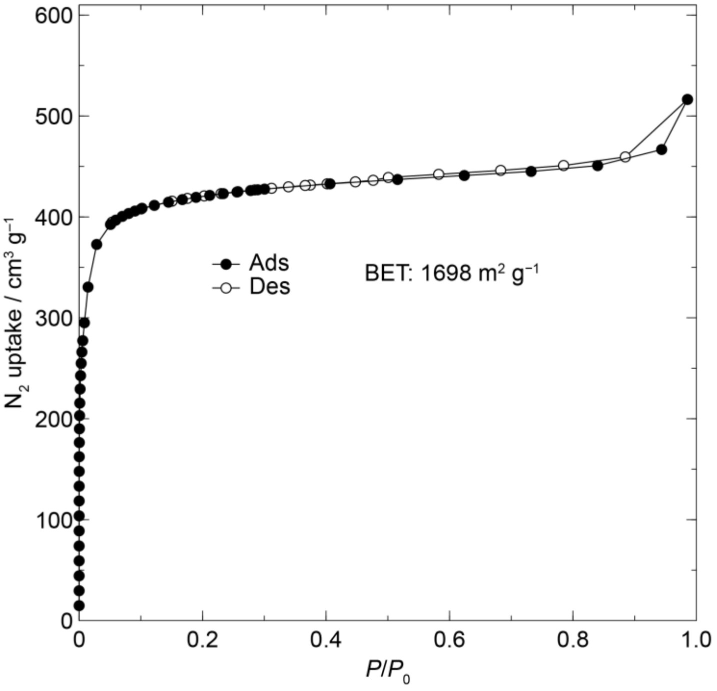

Figure S19. N_2 sorption isotherm at 77 K of activated COF-309. The filled and open circles represent the adsorption and desorption branches, respectively.

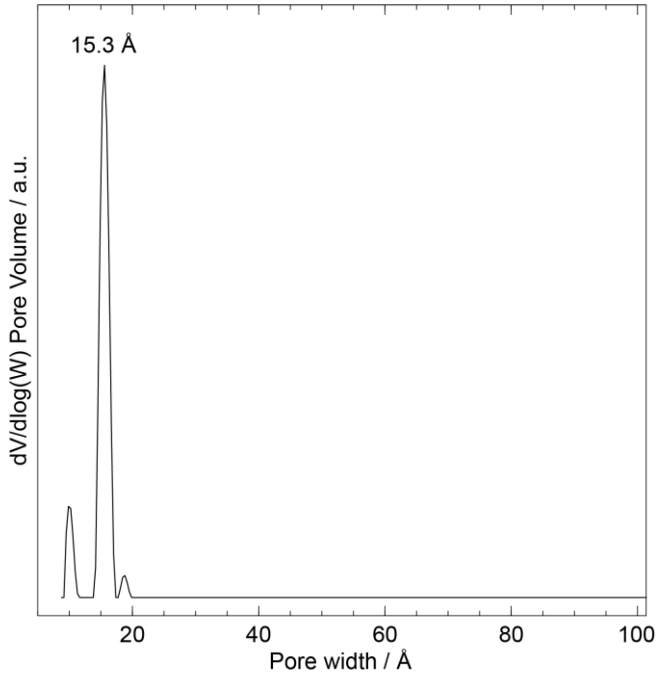

Figure S20. Pore size distributions of COF-309. The N_2-Cylindrical Pores-Oxide Surface model was used indicating a pore width of 15.3 Å which is similar to the model structure (14.7 Å).

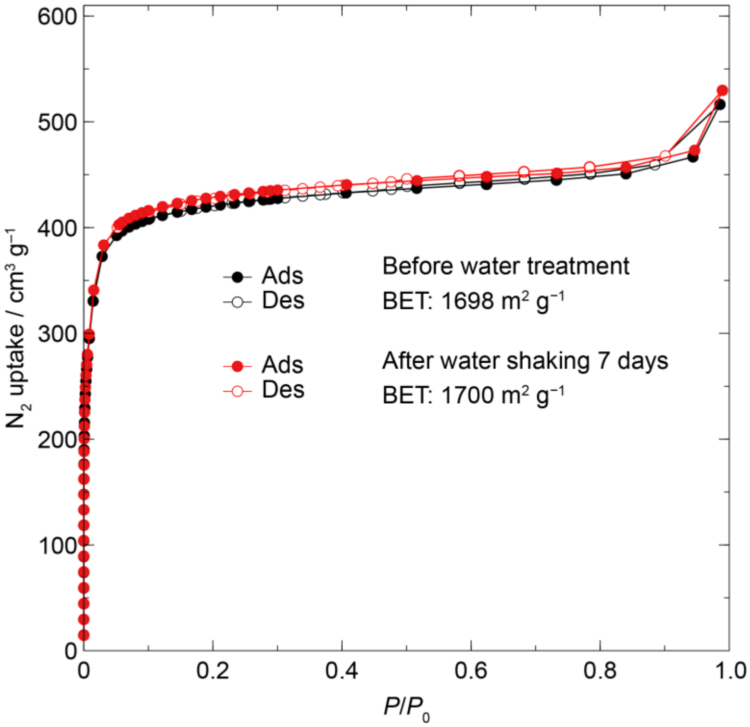

Figure S21. N_2 sorption analysis of COF-309 before and after water shaking at room temperature for 7 days. The filled and open circles represent the adsorption and desorption points, respectively. Procedure:

activated COF-309 (30 mg) was immersed in deionized water and shaken at 300 rpm for 7 d. After that, the COF solid was filtered and activated directly without solvent exchange.

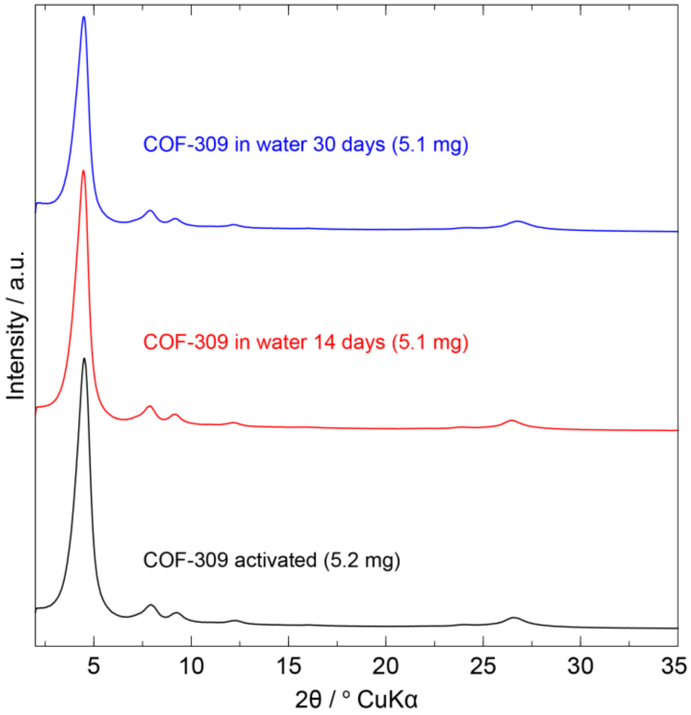

Figure S22. PXRD patterns of activated COF-309 and COF-309 after water treatment at room temperature for 14 and 30 days. Procedure: activated COF-309 (5.2 mg) was immersed in deionized water at room temperature. After that, the COF solid was filtered, roughly washed with acetone, and dried under vacuum at room temperature for 20 m before weighing.

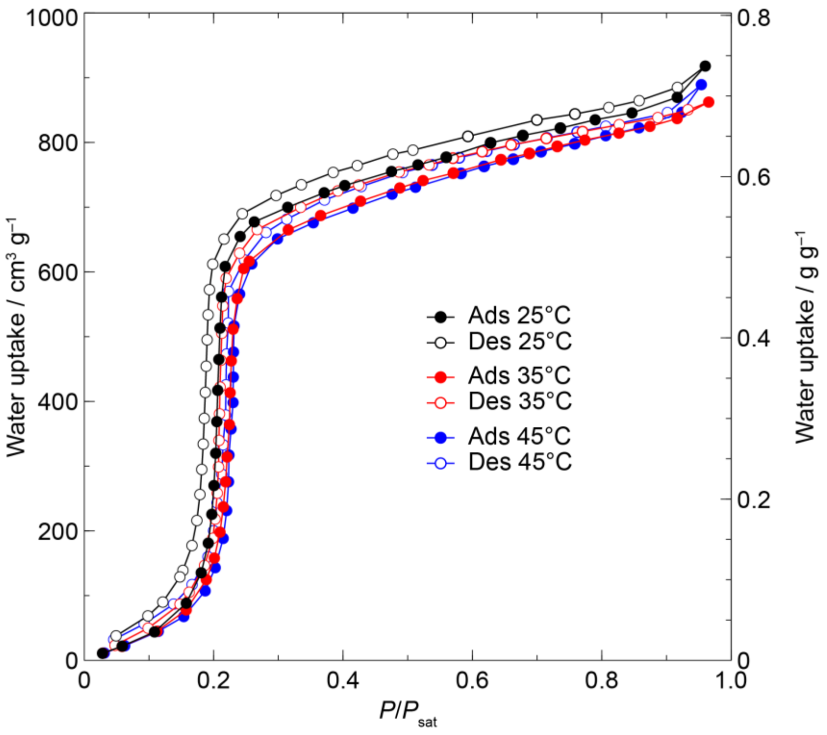

Figure S23. Water sorption analysis of COF-309 at different temperatures (25 °C, 35 °C, and 45 °C). *P*: Water vapor pressure. P_{sat}: The saturation vapor pressure at the respective temperature. The filled and open circles represent the adsorption and desorption points, respectively. *P*: Water vapor pressure. P_{sat}: The saturation vapor pressure at the respective temperature. The filled and open circles represent the adsorption and desorption points, respectively.

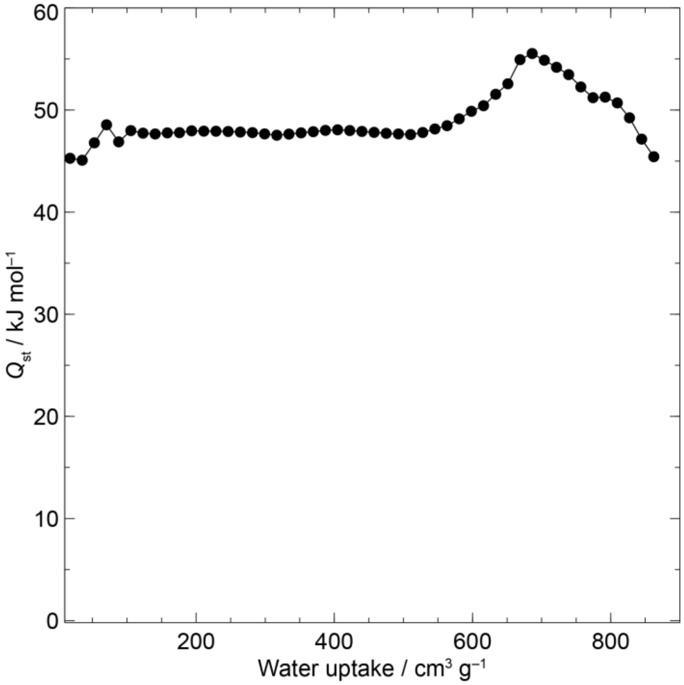

Figure S24. Isosteric heat of adsorption (Q_{st}) of COF-309 determined by the Clausius-Clapeyron relation applied to the water sorption isotherms measured at different temperatures (25 °C, 35 °C, and 45 °C).

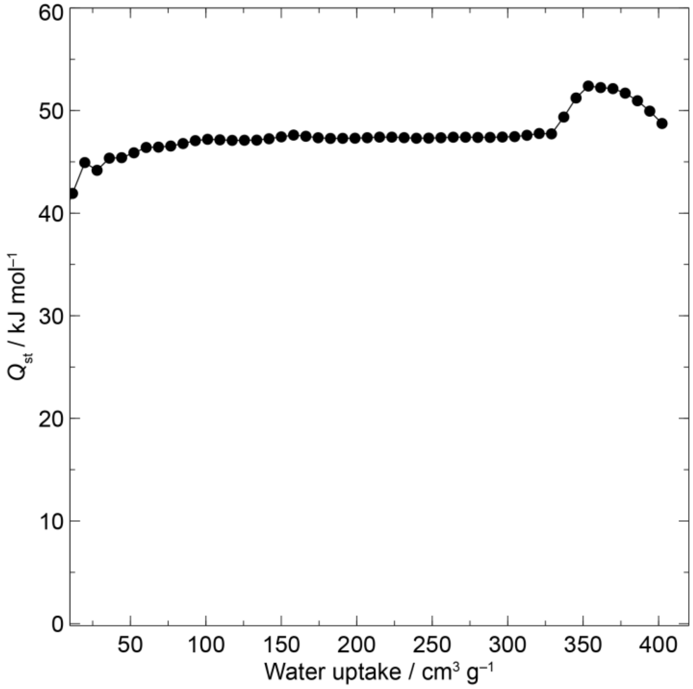

Figure S25. Isosteric heat of adsorption (Q_{st}) of HCOF-2 determined by the Clausius-Clapeyron relation applied to the water sorption isotherms measured at different temperatures (25 °C, 35 °C, and 45 °C).

Supporting Information Note 4 – Water Sorption Simulations

Monte Carlo simulation setup

Force-field-based Monte Carlo (MC) simulations were performed in the isobaric−isothermal (NpT) Gibbs ensemble to study the water adsorption in COFs using the Monte Carlo for Complex Chemical Systems-Minnesota software (MCCCSMN)[16]. The vapor-phase unary adsorption of water in the COFs was simulated using two simulation boxes: one for the sorbent phase and the second for water reservoir; in thermodynamic contact. A 2 × 2 × 5 supercell of every DFT-optimized COF crystal structure was used as sorbent and kept unchanged throughout the study. These structures were optimized in the presence of explicit water molecules, as described in previous sections, to account for structural deformation of the MOF under *wet* conditions; the explicit water molecules were removed before the isotherm simulations, and the COF framework was used without any further optimization. Such an approach has been previously used to simulate the water adsorption isotherms in MOF-303 to account for the structural deformations of the MOF in the presence of adsorbed water molecules.[17] The COF structure was held rigid throughout the simulation. In each adsorption simulation, rigid-body translational and rotational moves were performed on randomly selected water molecules, including swap moves between the two simulation boxes. Volume moves were performed only on the reservoir box to allow it to maintain the target pressure upon transfer of water molecules to the sorbent box. The Monte Carlo moves probabilities were randomly distributed in ratios of volume:swap:translational:rotational moves with values of 0.01:0.4:0.30:0.40 respectively.

The adsorption/desorption isotherm simulations were performed using 2000 water molecules at T = 298 K and P/P_{sat} = 0.01−1.0 (relative humidity, RH; saturation vapor pressure at 293 K: P_{sat} = 0.0419 atm). Simulations for the adsorption branches were started using an empty COF box without pre-adsorbed water molecules whereas the desorption branches were started from water-saturated COF structures. Each simulation equilibration was done for at least 60000 MC cycles (1 MC cycle = N MC moves where each N = number of water molecules in the system) with additional about 80000 MC cycles for the production period. The production period was divided into four equal blocks to determine the statistical uncertainties in the adsorbed water loading at each target pressure. The implemented force field was created with nonbonded Lennard-Jones (LJ) interaction parameters taken from the TraPPE force field[18], and the partial charges of every atom were

obtained from the DDEC6 charge scheme[19]. The intermolecular interactions of water molecules were described using the TIP4P water model[17,20] (P_{sat} = 4.54 ± 0.12 kPa at T = 298 K) for all COF structures. The Lorentz−Berthelot mixing rules were used to describe the COF−water LJ interactions. A spherical cutoff of 14 Å was used for truncating the pairwise Lennard-Jones and Coulomb interactions. Analytical tail corrections for the LJ interactions and the Ewald summation method for electrostatic interactions were employed.

Isotherm simulation details

In this section, the isotherm simulations are explained to give more details about the optimization step of the initial structures and the results of the simulation which show different behaviors based on the functional groups present in the COFs pores. Prior to the isotherm simulations, every COF structure is optimized with explicit water molecules added, to fill up the pore, in correspondence to the hydrophilic group of the COF pore using the R^2SCAN functional (see computational details section). HCOF-2 and HCOF-3 carry 12 water molecules per unit cell, and COF-309 carries 18. Each of these optimized structures are then used to perform the isotherm simulation after removal of the explicit water molecules. The modelled isotherms are in very good agreement with the measured isotherms; with the biggest deviation concerning the total water uptake of HCOF-2 most likely due to the presence of different staking modes in the experimental crystal structure that reduces the crystallinity level. The tautomerization occurring in HCOF-2 and HCOF-3 is a fundamental process that has to be considered in order to understand how the functional groups in the pore of the COF affect the water adsorption. It is noticeable that the structure of the COF with all the OH groups barely adsorb any water (orange line on the bottom side of the plot); however, the related tautomer, in which the proton from the OH group moved to the imine group, leaving a carbonyl group, shows water adsorption in agreement with the experimental measurements (red lines).

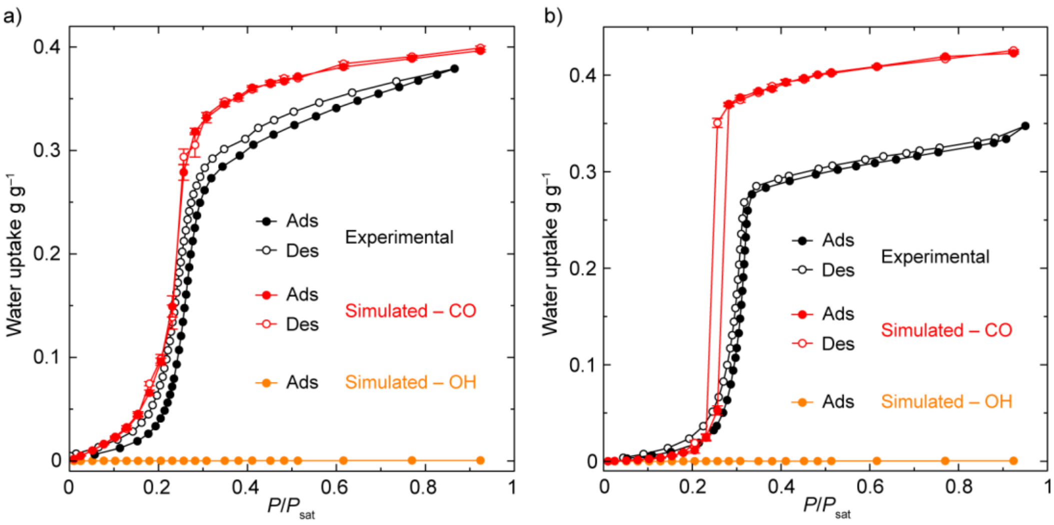

Figure S26. Simulated water isotherms for (a) HCOF-3 and (b) HCOF-2, with CO tautomerization closely match experimental data (error bars provided), while HCOFs with all OH do not show water sorption.

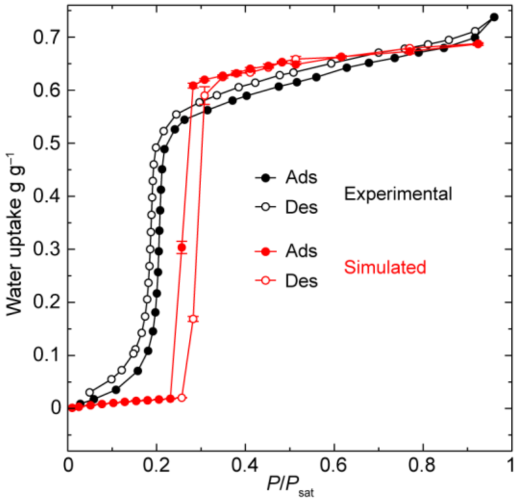

Figure S27. Simulated water isotherms for COF-309, with CO tautomerization, closely aligns with the simulated data.

The water binding starts at lower relative humidity, binding first to the carbonyl groups from which water cluster are built. The presence of hydroxyl groups does not seed water molecules, as found after geometry optimization (at the DFT R^2SCAN level), these water molecules are facilitating the tautomerization process, when OH groups are forced into the structure, the water molecules cluster among themselves rather than being adsorbed by the COF pore. The analysis of the absolute partial charges shows the O of the carbonyl groups to be –0.5 and the O of the hydroxyl –0.4. This slight

difference makes the carbonyl group slightly more hydrophilic than the hydroxyl, however this is not a definitive explanation.

To give a better explanation of the discrepancy in the adsorption results in the presence of enol-imine substituent groups, all functional groups present in the COF pores have been ranked in strength of water binding. This analysis points at rationalizing the difference between the simulated isotherms. The COF structures under consideration are: HCOF-2, HCOF-3, and COF-309. The hydrophilic functional groups present in the pores are carbonyl, imine, pyridine, and hydroxyl. In the next paragraphs the description of each of them, including their water binding energy is provided.

The first step is the calculation of the hydrogen-bond strength between two water molecules at the center of the COF ($E_{w\text{-}w}$). This energy value will then be used as a reference to rank the functional groups present in the COF pores: if their water-binding energy is lower (more positive relative energy change) than $E_{w\text{-}w}$, most likely, they will not show favorable nucleation and adsorption due to the preference of the waters to cluster among themself; on the other hand, the functional groups that result in a water binding energy higher (more negative relative energy change) that $E_{w\text{-}w}$ will show good nucleation and thus good water adsorption. $E_{w\text{-}w}$ was calculated as the difference in electronic energy of a COF with one and with two water molecules placed in the center of the COF pore (Figure S28). The resulting value is –9.4 kcal mol^{-1} (SCAN-D3BJ). As a reference the hydrogen bond strength of a water dimer in vacuum is –6.6 kcal mol^{-1} (SCAN-D3BJ). The value –9.4 kcal mol^{-1} thus can be used as a threshold to rank each functional group of the COF pore and their higher (more negative relative energy change) or lower (more positive relative energy change) water binding strength.

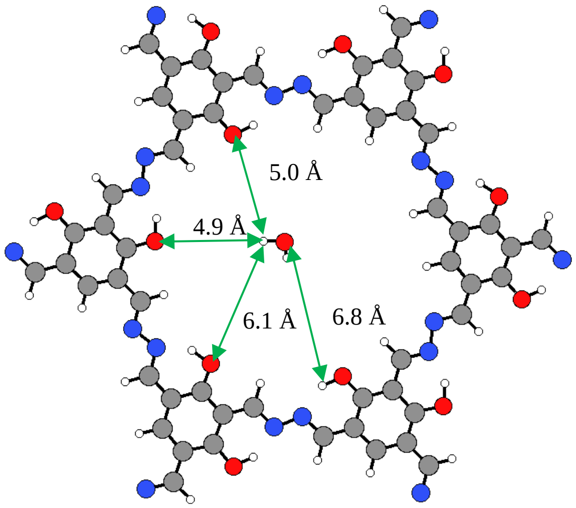

Figure S28. Water molecule in the center of the pore of HCOF-2. Colorcode = C: gray; O: red; N: blue; H: white.

The first functional group that has been considered is the CO with a water binding energy of ca. –16 kcal/mol. This is the highest binding energy among all functional groups and for this reason CO is the first group to coordinate to water. At low pressure the water molecule bridge-binds two CO oxygens belonging to two subsequent layers with a CO—H(water) distance of 1.9 Å (Figure S29 left). This bridge can be assumed to be an initial water nucleation which is then broken with the increase of the pressure when each carbonyl group binds to one water molecule with a CO—H(water) distance of 1.9 Å (Figure S29 right), and from these water molecules the pore will continue to be filled while increasing the vapor pressure.

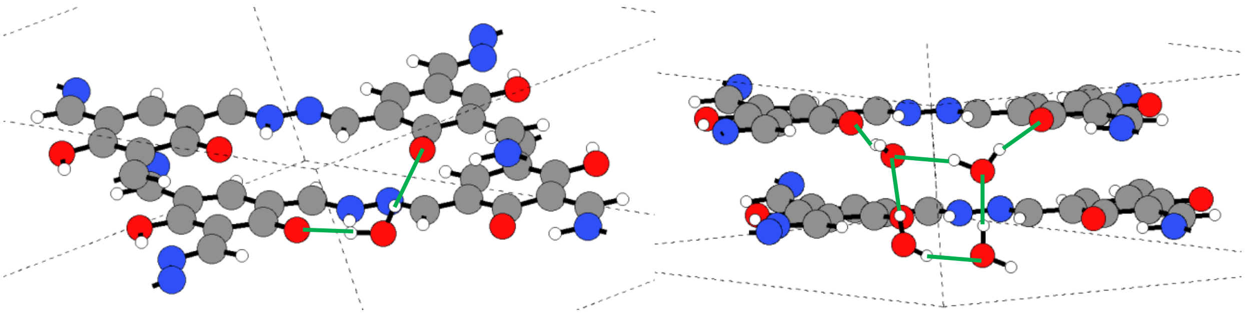

Figure S29. Initial water bridging CO groups at lower and higher partial pressures. Colorcode = C: gray; O: red; N: blue; H: white.

The imine group has a water binding energy of –10.3 kcal mol^{-1}, similar to the pyridine moiety value of –10.8 kcal mol^{-1}. Both nitrogen atoms of these functional groups coordinate water through

the lone pair using the sp^2 orbital. The electronic water binding energy to both imine and pyridine is therefore 1 kcal/mol higher (more negative relative energy change) than the $E_{w\text{-}w}$ (–9.4 kcal mol^{-1}). This could be the explanation for the slightly higher vapor pressure needed for the water to coordinate to the imine group, which, during the simulation, happens after the water coordinates to each of the CO groups, as found in both DFT and MC simulation.

All the functional groups considered so far show a water binding value higher than $E_{w\text{-}w}$ (more negative relative energy change). The situation changes only for the OH group. This group together with the neighboring imine group is responsible for the tautomerization which is caused by the hydrogen bond interaction between them that facilitates the proton transfer in presence of water, as shown in previous DFT results. The O—H distance is 1.0 Å, and the N—H distance is 1.6 Å in the β-keto enamine. In its tautomeric form the N—H distance becomes 1.1 Å and the O—H 1.7 Å. These values (valid for both HCOF-2 and HCOF-3) confirm that these functional groups are interacting via hydrogen bond in both tautomers. The water binding energy with the OH group is –6.1 kcal mol^{-1} which is lower (more positive relative energy change) than $E_{w\text{-}w}$ and this is in line with the lack of water adsorption of this tautomer. The position of the water molecule when interacting with the OH group is in between the O of the OH and the H of the CH located in β-position of the imine group (Figure S30).

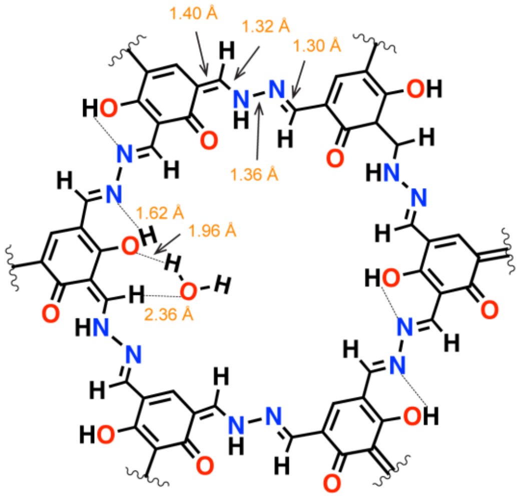

Figure S30. Bond lengths and position of water when interacting with the OH group of HCOF-2.

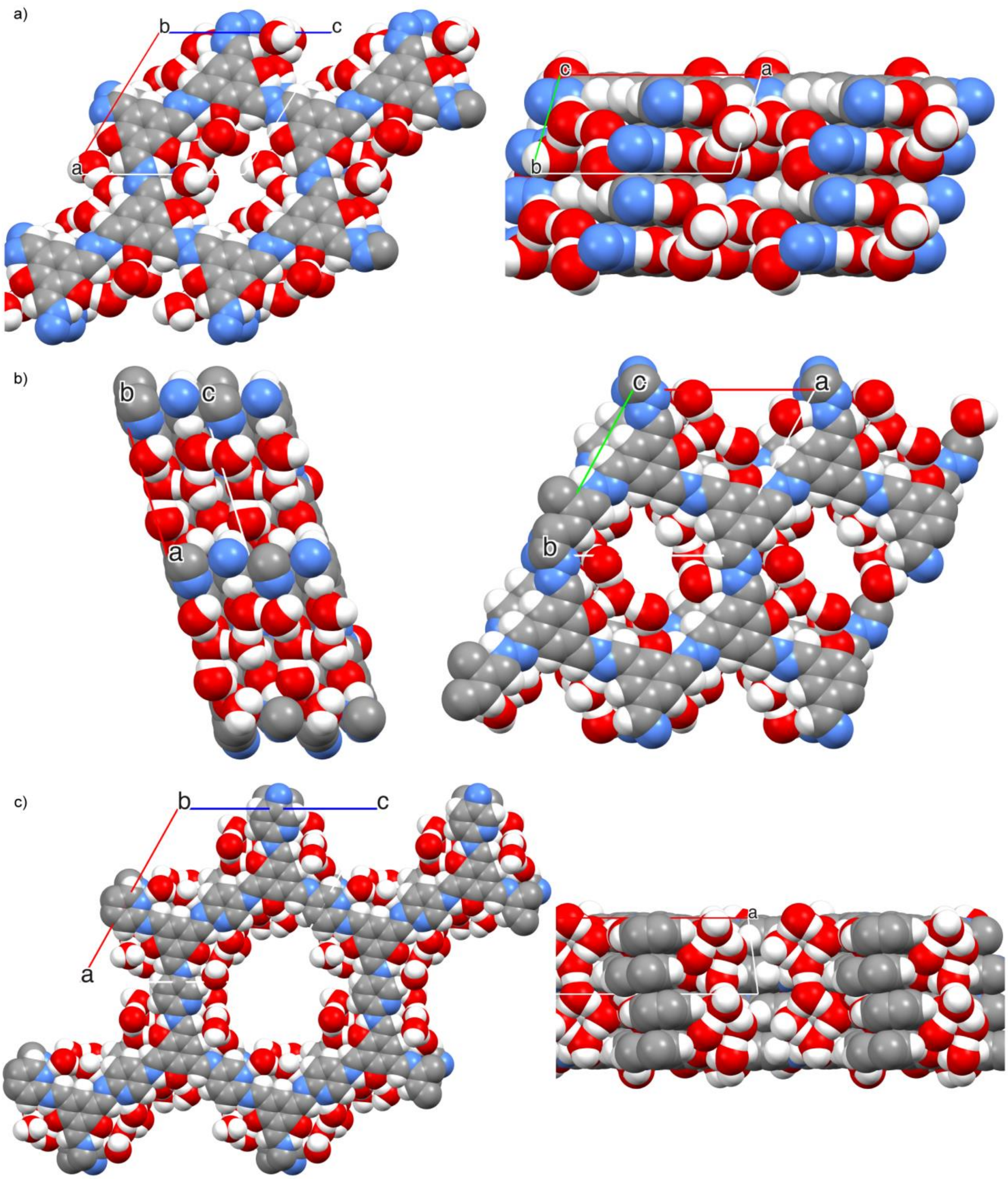

Figure S31. Arrangement of water molecules in the pores of (a) HCOF-2, (b) HCOF-3, and (c) COF-309. Water preferably forms channels along the pores of COFs (projected *c* axis in HCOF-2, *b* axis in HCOF-3, and *c* acis in COF-309).

Force field details

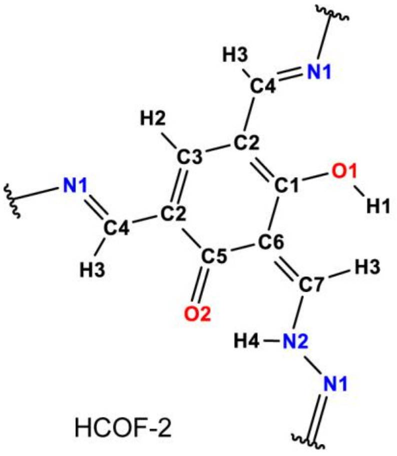

HCOF-2

HCOF-2				
type	ε/kB [K]	σ [Å]	q [e]	Molecule name
C1	30.7	3.60	0.332	phenol
C2	30.7	3.60	–0.112	phenol
C3	30.70	3.60	–0.086	phenol
C4	30.70	3.60	0.075	pyrazole
C5	30.00	3.45	0.363	*p*-benzoquinone
C6	30.70	3.60	–0.245	phenol
C7	30.70	3.60	0.120	pyrazole
N1	57.00	3.20	–0.205	pyrazole
N2	141.00	3.40	–0.084	pyrazole
O1	118.00	3.04	–0.433	phenol
O2	90.00	2.95	–0.490	*p* -benzoquinone
H1	12.00	0.50	0.276	phenol
H2	25.45	2.36	0.108	phenol
H3	25.45	2.36	0.102	pyrazole
H4	12.00	0.50	0.319	pyrrole

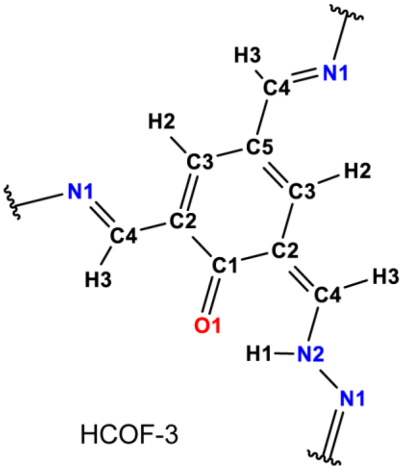

HCOF-3

HCOF-3				
type	ε/kB [K]	σ [Å]	q [e]	Molecule name
C1	30.00	3.46	0.337	*p*-benzoquinone
C2	30.70	3.60	–0.090	phenol
C3	30.70	3.60	–0.107	phenol
C4	30.70	3.60	0.076	pyrazole
C5	30.70	3.60	–0.024	*p*-benzoquinone
N1	57.00	3.20	–0.201	pyrazole
N2	141.00	3.40	–0.073	pyrazole
O1	90.00	2.95	–0.491	*p*-benzoquinone
H1	12.00	0.50	0.269	phenol
H2	25.45	2.36	0.107	phenol
H3	25.45	2.36	0.095	pyrazole

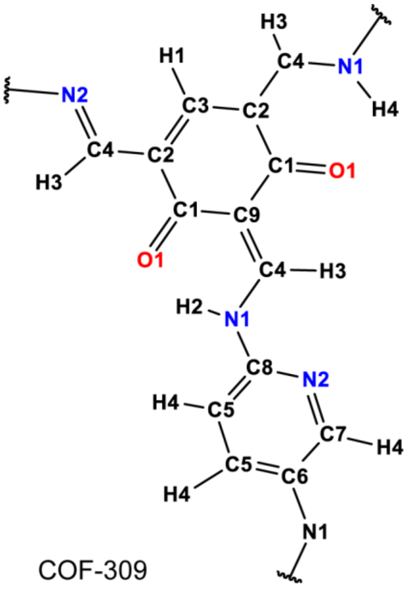

COF-309				
type	ε/kB [K]	σ [Å]	q [e]	Molecule name
C1	30.00	3.45	0.373	*p*-benzoquinone
C2	30.70	3.60	–0.122	*p*-benzoquinone
C3	30.70	3.60	–0.062	phenol
C4	30.70	3.60	0.144	pyridine
C5	30.70	3.60	–0.166	pyridine
C6	55.00	3.20	0.159	purine
C7	30.70	3.60	0.036	pyridine
C8	55.00	3.20	0.357	purine
C9	30.70	3.60	–0.264	*p*-benzoquinone
O1	90.00	2.95	–0.492	*p*-benzoquinone
N1	141.00	3.40	–0.276	pyrrole
N2	57.00	3.20	–0.355	pyridine
H1	25.45	2.36	0.113	phenol
H2	12.00	0.50	0.288	pyrrole
H3	25.45	2.36	0.091	pyrrole
H4	25.45	2.36	0.117	pyridine

Figure S32. Chemical structures and force field details of MC simulations for HCOF-2, HCOF-3, and COF-309.

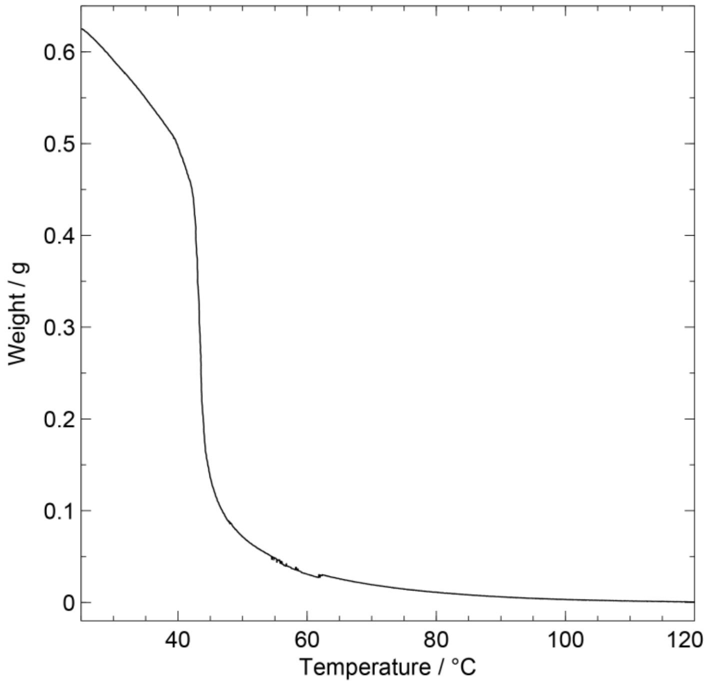

Figure S33. Water isobaric profile of COF-309 showing that 60°C is the optimal temperature for water desorption of COF-309. Experiment conditions: 1.70 kPa, swing temperature range between 30 °C and 60 °C.

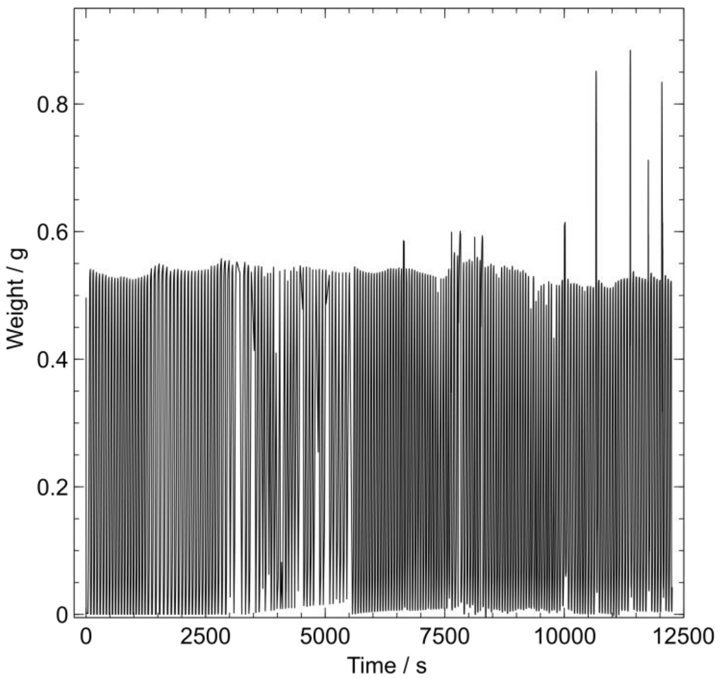

Figure S34. Water cycling stability test of COF-309 at constant water vapor pressure (1.7 kPa). Over 170 adsorption-desorption cycles were conducted at 30 °C (corresponding to 40% relative humidity (RH)) and 60 °C (corresponding to 10% RH), respectively. Fluctuation was found in some cycles but overall, the working capacity of COF-309 retains 0.52 g g^{-1} after 170 adsorption-desorption cycles.

Supporting Information Note 5 – Adsorptive Site Calculation

The number of adsorptive sites calculation for COFs

Water binding energy calculations done with the functional SCAN-D3BJ on top of optimized structures with R^2SCAN-D3BJ functional in the periodic system (see previous section for full details) indicate that water molecules interact with C=O functional groups with a binding energy of $\Delta E_{CO\text{-}w} = -16$ kcal mol^{-1}, which is stronger than that of water–water interaction ($\Delta E_{w\text{-}w} = -9.4$ kcal mol^{-1}, SCAN-D3BJ functional). Therefore, C=O will always be considered as one adsorptive site. A N-based functional group (pyridine or imine) interacts with water with no obstruction and will be considered as one adsorptive site, with less negative binding energies of –10.3 kcal mol^{-1}, and –10.8 kcal mol^{-1} imine and pyridine moiety respectively (SCAN-D3BJ). Based on these criteria, COF-309 has 18 adsorptive site per one unit cell.

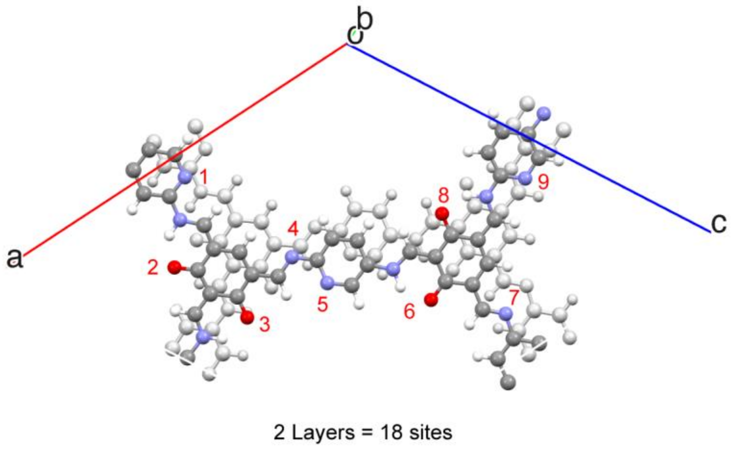

Figure S35. One unit cell of COF-309 contains 2 layers with a total of 18 adsorptive sites.

In HCOFs (e.g., HCOF-3), C=O is counted one adsorptive site. Two N atoms in proximity (<3.3 Å) are counted one adsorptive site.

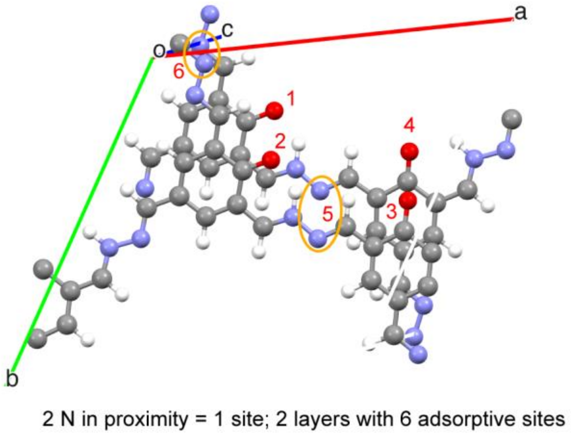

Figure S36. One unit cell of HCOF-3 contains 2 layers with a total of 6 adsorptive sites.

The proximity consideration is also applied to COFs consisted of imine or hydrazine groups and no C=O groups, for example AB-COF and Py-COF-2D. In the case of COF-432, the pore size of this structure is small (8 Å), C=N functionalities are not close with each other. Therefore, we consider 4 N atoms per one pore as one adsorptive site.

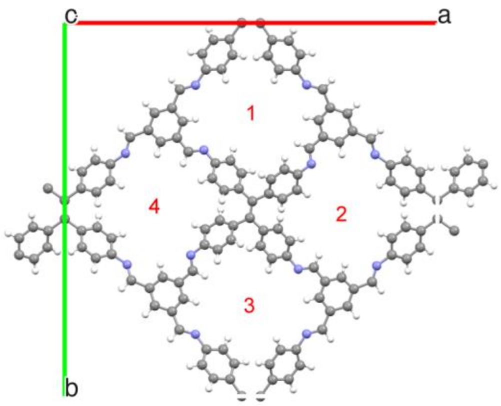

Figure S37. One unit cell of COF-432 contains 4 layers (only one is presented here for simplification) with a total of 16 adsorptive sites.

Below are some general rules for counting adsorptive sites in a COF/MOF structure:

Rule 1: Polar groups such as CO, OH, NH_2, and pyridine account for water adsorptive sites.

Rule 2: If OH is covered by steric hindrance or bonded by an H-bond, it will be excluded.

Rule 3: If two polar groups (e.g., imine) are in proximity, only one site is counted.

Rule 4: It is necessary to perform calculations to determine the binding energy between water and the functional group. Only strong interactions (larger than the water–water binding energy) will be counted.

Table S2 describes the water adsorption properties and the number of adsorptive sites calculated for each COF.

Table S2. Water sorption properties and structural features of COFs

COF	Theoretical BET area / $m^2\,g^{-1}$	RH onset at 25 °C / %	Number of adsorptive sites per unit cell	$\overline{\Delta H_{ads}}$ / kJ mol^{-1}	Functionality
AB-COF[21,22]	1962	24.7	6	-49.25	Hydrazine<br>2 Layers, 12 N pairs
HCOF-2	1148	30.9	6	-47	β-Ketoenamine, hydrazine<br>2 Layers, 4 CO, 4 N pair
HCOF-3	1212	26.1	6	-48	β-Ketoenamine, hydrazine<br>2 Layers, 4 CO, 4 N pairs
COF-309	1768	20.4	18	-48.1	β-Ketoenamine, imine<br>2 Layers, 8 CO, 10 CN, 10 pyridine
BDA-DHTA[4,23]	1610	25.5	6	-47.5	β-Ketoenamine, imine<br>1 Layer, 4 CO, 2 CN
COF-432[24]	1000	34	16	-48	Imine<br>4 Layers, 4 water seedings/layer
Py-COF-2D[22]	1980	56	8	-45	Hydrazine<br>2 Layers, 18 N pairs
COF-ok[25]	1959	30.4	8	-49.5	*o*-Ketoenamine<br>1 Layer, 8 CO

The number of adsorptive sites calculation for MOFs

The adsorption properties of Al-based MOFs which have been previously investigated for their water adsorption behavior were included in this analysis. **Table S3** describes the water adsorption properties and the number of adsorptive sites calculated for each MOF.

Table S3. Water sorption properties and structural features of MOFs

MOF	Theoretical BET area / $m^2 g^{-1}$	RH onset at 25 °C / %	Number of adsorptive sites per unit cell	$\overline{\Delta H_{ads}}$ / kJ mol^{-1}	Functionality
MOF-303[26]	1355	11.8	12	-52.8	Pyrazole linker and μ_2-OH rod functionalities<br><br>4 site I: -NH_{linker}, -N_{linker}, μ_2-OH<br><br>4 site II: -NH_{linker}, -N_{linker}<br><br>4 site III: μ_2-OH
MOF-333[26]	1321	22.3	8	-50.4	Furan linker and μ_2-OH rod functionalities<br><br>8 site I: μ_2-OH, $O_{carboxylate}$<br><br>No strong interactions were observed with O_{furan}
MOF-LA2-1(pyrazole)[27]	1892	26	12	-51.0	Pyrazole linker and μ_2-OH rod functionalities<br><br>4 site I: -NH_{linker}, -N_{linker}, μ_2-OH<br><br>4 site II: -NH_{linker}, -N_{linker}<br><br>4 site III: μ_2-OH
LAMOF-2[28]	1691	36	16	-47.9	Thiophene linker and μ_2-OH rod functionalities<br><br>16 site I: μ_2-OH, $O_{carboxylate}$<br><br>No strong interactions were observed with S_{furan}
LAMOF-3[28]	1423	45	8	-46.5	Thiophene linker and μ_2-OH rod functionalities<br><br>8 site I: μ_2-OH, $O_{carboxylate}$

					No strong interactions were observed with S_{furan}
LAMOF-4[28]	1847	40	16	-48.7	Furan linker and μ_2-OH rod functionalities<br>16 site I: μ_2-OH, $O_{carboxylate}$<br>No strong interactions were observed with O_{furan}
LAMOF-5[28]	1387	13	32	-53.6	Furan linker and μ_2-OH rod functionalities<br>16 site I: μ_2-OH, $O_{carboxylate}$<br>16 site I: O_{furan}, $O_{carboxylate}$
MOF-LA2-1(furan)[28,29]	1209	14	32	-53.0	Furan linker and μ_2-OH rod functionalities<br>16 site I: μ_2-OH, $O_{carboxylate}$<br>16 site I: O_{furan}, $O_{carboxylate}$
MOF-LA2-2(furan)[29]	1550	30	32	-50.0	Furan linker and μ_2-OH rod functionalities<br>16 site I: μ_2-OH, $O_{carboxylate}$<br>16 site I: O_{furan}
CAU-23[30]	1250	29	16	-48.2	Thiophene linker and μ_2-OH rod functionalities<br>16 site I: μ_2-OH, $O_{carboxylate}$<br>No strong interactions expected with S_{furan}
MIL-160[31]	1070	8	32	-52.0	Furan linker and μ_2-OH rod functionalities<br>16 site I: μ_2-OH, $O_{carboxylate}$<br>16 site I: O_{furan}, $O_{carboxylate}$

There is another way to render i_H dimentionless, which we use a relative i_H – a ratio between the given i_H to a standard i_H ($i_{\mathrm{H_{std}}}$).We selected MOF-303 ($RH_{\mathrm{onset_{std}}}$ = 11.8%) and BDA-DHTA ($RH_{\mathrm{onset_{std}}}$ =

25.5%) as the standard water-harvesting MOF and COF, respectively, to determine the relative i_H for various MOFs and COFs.

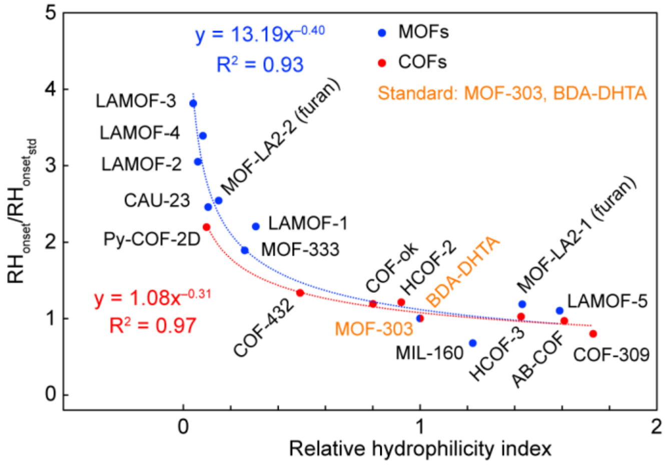

Figure S38. Correlation between $\frac{RH_{\text{onset}}}{RH_{\text{onset}_{\text{std}}}}$ and the relative i_H of microporous COFs (red) and MOFs (blue). MOF-303 and BDA-DHTA were chosen as standard water-harvesting MOF and COF, respectively, for the relative i_H calculation. As the relative i_H increases, the onset position (RH_{onset}) of the water adsorption isotherms of COFs/MOFs decreases (i.e., $\frac{RH_{\text{onset}}}{RH_{\text{onset}_{\text{std}}}}$ reduces), shifting to the lower relative humidity.

Below is a step-by-step process for estimating the onset position (RH_{onset}) of a reticular structure using the i_H equation proposed in the manuscript [equations (1) and (2)]. For this example, we use MOF-801,[32] which was not included in the fitting shown in Figure 4 of the main text.

- MOF-801 has two primary adsorptive sites (sites I and II) per each tetrahedral cage. Sites III, IV, and V involve hydrogen bonding between water molecules at high relative humidity and are not considered in calculation of the number of adsorptive sites (N_{ads}).
- MOF-801 with **fcu** topology contains 8 tetrahedral cages per one unit cell. Therefore, N_{ads} is 16. Theoretical surface area of MOF-801 is about 990 m^2 g^{-1}, and the average isosteric heat of water adsorption ($\overline{\Delta H_{\text{ads}}}$) is -60 kJ mol^{-1}.
- Using equation (1), i_H of MOF-801 is calculated to be 97.682.
- By applying the fitting equation based on equation (2) for MOFs, y = $32.80x^{-0.39}$, where y is RH_{onset} and x corresponds to i_H, RH_{onset} of MOF-801 is estimated to be 5.5% RH. The experimental value is 9% RH.

Supporting Information Note 6 – Q_{st} Estimation

Estimation of isosteric heat of adsorption (Q_{st}) using the electronic energies of H-bonds formed between water molecules and various water-adsorbing functional groups

In the specific case of adsorbed water clusters, each water molecule can form 4 H-bonds either with water-adsorbing functional groups or neighboring water molecules. Such water clusters also have a density similar to that of ice ($\rho_{cluster} \approx \rho_{ice} = 0.9167$ g cm^{-3}).

The average binding energy (E_{Avg}, in kJ mol^{-1}) is calculated following the OLSA equation (OLSA: Omar-Lac Ha-Saumil-Ali):

$$E_{Avg} = \frac{\sum E_f + \frac{1}{2}(4N_{H_2O} - N_f)E_{H_2O-H_2O}}{N_{H_2O}}$$

E_f is the electronic energy of a H-bond formed between one water molecule and a specific water-adsorbing functional group (in kJ mol^{-1}).

N_f is the total number of water-adsorbing functional groups in one unit cell of the MOF/COF.

$E_{H_2O-H_2O}$ is the electronic energy of a single water-water interaction (20 kJ mol^{-1}).

N_{H_2O} is the number of water molecules per one unit cell of the MOF/COF, estimated using the following equation:

$$N_{H_2O} = \frac{\rho_{cluster} \times V_{Pore}}{MW_{H_2O}}$$

$\rho_{cluster} \approx \rho_{ice} = 0.9167$ g cm^{-3}

V_{Pore} is the theoretical pore volume of the MOF/COF per one unit cell (cm^3 mol^{-1})

MW_{H_2O} is the molecular weight of water (18.0153 g mol^{-1}).

The E_f values (**Table S5**) for the H-bond between a water molecule and different linker functional groups are computed by performing a potential energy scan between a water molecule and an isolated linker in the *Gaussian 16* software[33]. The isolated linkers were capped with Na ions, representative the metal-organic linkages, to ensure charge neutrality. Specifically, the distance of the O atom of the water molecule from the target functionality was stepped in fine intervals of 0.10 Å between 1.5 Å to 3.0 Å, followed by larger intervals of 0.25 Å between 3.0 Å and 6.5 Å. The E_f value was then calculated as the difference between the most stable configuration and the

last configuration of the scan. The M06-L density functional[34] was used along with the def2TZVP basis set[35,36] for all the atoms. For linkers with more than one adsorbing functional group, the potential energy scans were performed for each functional group by constraining a straight H-bond angle ($\angle X_{donor}$-H···$Y_{acceptor}$ = 180°) in the plane of the aromatic core of the linker.

Table S4. Calculated electronic energies of the H-bond of a water molecule with various water-adsorbing functional groups in MOFs

Linker	Functional groups	Absolute electronic energy (Hartree)	E_f (kJ mol^{-1})
3,5-pyrazoledicarboxylate	-N	–1003.490993	–41.3
	-NH	–1003.487905	–33.1
2,4-furandicarboxylate	-O	–1007.303978	–15.1
2,5-furandicarboxylate	-O	–1007.310972	–29.2
2,4-thiophenedicarboxylate	-S	–1330.275791	–9.9
2,5-thiophenedicarboxylate	-S	–1330.277991	–11.5
2,4-pyrroledicarboxylate	-NH	–987.4581832	–42.2
2,5-pyrroledicarboxylate	-NH	–987.4628675	–37.4
2,4-imidazoledicarboxylate	-N	–1003.506400	–39.9
2,5-imidazoledicarboxylate	-N	–1003.512610	–34.7
	-NH	–1003.512571	–34.8
3,5-triazoledicarboxylate	-N(2)	–1019.541243	–38.5
	-N(4)	–1019.542348	–39.8
	-NH	–1019.540230	–36.8
2,5-triazoledicarboxylate	-N	–1019.538595	–31.6
	-NH	–1019.538344	–33.3
3,5-pyridinedicarboxylate	-N	–1025.567588	–31.4

Table S5. Estimated electronic energy of H-bonds formed between water molecules and various water-adsorbing functional groups in MOFs and COFs

MOF/COF	Functional groups	N_f	Total E_f (kJ mol^{-1})	N_{H_2O}	Average Q_{st} (exp) (kJ mol^{-1})	E_{Avg} (est) (kJ mol^{-1})
MOF-303	Pyrazole	24	–987.2	40.2	–52.8	–60.3
MOF-333	Furan	16	–512.8	40.5	–50.4	–50.5
MOFLA2-1 pyrazole/LAMOF-1	Pyrazole	24	–987.2	61.1	–51	–54.0
LAMOF-2	Thiophene	32	–942.4	119.5	–47.9	–47.1
LAMOF-3	Thiophene	16	–484	49.5	–46.5	–48.4
LAMOF-4	Furan	32	–1025.6	118.0	–48.7	–47.8
MOF-LA2-1(furan)	Furan	32	–1251.2	79.0	–53	–53.6
MOF-LA2-2(furan)	Furan	32	–1251.2	121.6	–50	–49.5
CAU-23	Thiophene	32	–968	83.7	–48.2	–49.6
MIL-160	Furan	32	–1251.2	64.2	–52	–56.3
AB-COF	Imine	12	–357.6	25.0	–49.25	–47.0
HCOF-2	β-Ketoenamine, imine	12	–367.2	16.1	–47	–51.9[a]
HCOF-3	β-Ketoenamine, imine	12	–362.4	15.1	–48	–51.5[a]
COF-309	β-Ketoenamine, imine, pyridine	18	–525	54.0	–48.1	–46.7
NTU-BDA-DHTA	β-Ketoenamine, imine	6	–176.6	23.4	–47.5	–45.9
COF-432	Imine	48	–1430.4	124.5	–48	–46.0
Py-COF-2D	Imine	16	–476.8	111.7	–45	–43.5
COF-ok	o-Ketoenamine	8	–248	63.2	–49.5	–43.9

[a]Discrepancy between E_{Avg} and Q_{st} in HCOFs is attributed to the small pores of the two COFs which leads to the fact that some water molecules have fewer than 4 H-bonds.

References

(1) Ai, L.; Li, W.; Wang, Q.; Cui, F.; Jiang, G. Harnessing Keto-Enol Tautomerism to Modulate β-Ketoenamine-Based Covalent Organic Frameworks for Visible-Light-Driven CO2 Reduction. *ChemCatChem* **2022**, *14*, e202200935.

(2) Haldar, S.; Chakraborty, D.; Roy, B.; Banappanavar, G.; Rinku, K.; Mullangi, D.; Hazra, P.; Kabra, D.; Vaidhyanathan, R. Anthracene-Resorcinol Derived Covalent Organic Framework as Flexible White Light Emitter. *J. Am. Chem. Soc.* **2018**, *140*, 13367–13374.

(3) Kandambeth, S.; Mallick, A.; Lukose, B.; Mane, M. V.; Heine, T.; Banerjee, R. Construction of Crystalline 2D Covalent Organic Frameworks with Remarkable Chemical (Acid/Base) Stability via a Combined Reversible and Irreversible Route. *J. Am. Chem. Soc.* **2012**, *134*, 19524–19527.

(4) Wang, H.; Qian, C.; Liu, J.; Zeng, Y.; Wang, D.; Zhou, W.; Gu, L.; Wu, H.; Liu, G.; Zhao, Y. Integrating Suitable Linkage of Covalent Organic Frameworks into Covalently Bridged Inorganic/Organic Hybrids toward Efficient Photocatalysis. *J. Am. Chem. Soc.* **2020**, *142*, 4862–4871.

(5) BIOVIA. 2019, p Materials Studio, San Diego, Dassault Systèmes.

(6) Kresse, G.; Furthmüller, J. Efficiency of Ab-Initio Total Energy Calculations for Metals and Semiconductors Using a Plane-Wave Basis Set. *Comput. Mater. Sci.* **1996**, *6*, 15–50.

(7) Furness, J. W.; Kaplan, A. D.; Ning, J.; Perdew, J. P.; Sun, J. Accurate and Numerically Efficient R2SCAN Meta-Generalized Gradient Approximation. *J. Phys. Chem. Lett.* **2020**, *11*, 8208–8215.

(8) Grimme, S.; Antony, J.; Ehrlich, S.; Krieg, H. A Consistent and Accurate Ab Initio Parametrization of Density Functional Dispersion Correction (DFT-D) for the 94 Elements H-Pu. *J. Chem. Phys.* **2010**, *132*, 154104.

(9) Kresse, G.; Joubert, D. From Ultrasoft Pseudopotentials to the Projector Augmented-Wave Method. *Phys. Rev. B* **1999**, *59*, 1758–1775.

(10) Blöchl, P. E. Projector Augmented-Wave Method. *Phys. Rev. B* **1994**, *50*, 17953–17979.

(11) Sun, J.; Ruzsinszky, A.; Perdew, J. P. Strongly Constrained and Appropriately Normed Semilocal Density Functional. *Phys. Rev. Lett.* **2015**, *115*, 36402.

(12) Becke, A. D. Density‐ functional Thermochemistry. III. The Role of Exact Exchange. *J. Chem. Phys.* **1993**, *98*, 5648–5652.

(13) Lee, C.; Yang, W.; Parr, R. G. Development of the Colle-Salvetti Correlation-Energy Formula into a Functional of the Electron Density. *Phys. Rev. B* **1988**, *37*, 785–789.

(14) Vosko, S. H.; Wilk, L.; Nusair, M. Accurate Spin-Dependent Electron Liquid Correlation Energies for Local Spin Density Calculations: A Critical Analysis. *Can. J. Phys.* **1980**, *58*, 1200–1211.

(15) Stephens, P. J.; Devlin, F. J.; Chabalowski, C. F.; Frisch, M. J. Ab Initio Calculation of Vibrational Absorption and Circular Dichroism Spectra Using Density Functional Force Fields. *J. Phys. Chem.* **1994**, *98*, 11623–11627.

(16) Siepmann, J. I.; Martin, M. G.; Chen, B.; Wick, C.; Stubbs, J. M.; Potoff, J. J.; Eggimann, B. L.; McGrath, M. J.; Zhao, X. S.; Anderson, K. E.; Rafferty, J. L.; Rai, N.; Maerzke, K. A.; Keasler, S. J.; Bai, P.; Fetisov, E. O.; Shah, M. S.; Chen, Q. P.; DeJaco, R. F.; Chen, J. L.; Xue, B.; Bunner, C.; Sun, Y. Z. S.; Josephson, T. R. Monte Carlo for Complex Chemical Systems. *Minnesota, version 20.2; Univ. Minnesota Minneapolis, MN* **2020**.

(17) Chheda, S.; Jeong, W.; Hanikel, N.; Gagliardi, L.; Siepmann, J. I. Monte Carlo Simulations of

Water Adsorption in Aluminum Oxide Rod-Based Metal–Organic Frameworks. *J. Phys. Chem. C* **2023**, *127*, 7837–7851.

(18) Rai, N.; Siepmann, J. I. Transferable Potentials for Phase Equilibria. 9. Explicit Hydrogen Description of Benzene and Five-Membered and Six-Membered Heterocyclic Aromatic Compounds. *J. Phys. Chem. B* **2007**, *111*, 10790–10799.

(19) Manz, T. A.; Limas, N. G. Introducing DDEC6 Atomic Population Analysis: Part 1. Charge Partitioning Theory and Methodology. *RSC Adv.* **2016**, *6*, 47771–47801.

(20) Jorgensen, W. L.; Chandrasekhar, J.; Madura, J. D.; Impey, R. W.; Klein, M. L. Comparison of Simple Potential Functions for Simulating Liquid Water. *J. Chem. Phys.* **1983**, *79*, 926–935.

(21) Stegbauer, L.; Hahn, M. W.; Jentys, A.; Savasci, G.; Ochsenfeld, C.; Lercher, J. A.; Lotsch, B. V. Tunable Water and CO2 Sorption Properties in Isostructural Azine-Based Covalent Organic Frameworks through Polarity Engineering. *Chem. Mater.* **2015**, *27*, 7874–7881.

(22) Nguyen, H. L.; Gropp, C.; Hanikel, N.; Möckel, A.; Lund, A.; Yaghi, O. M. Hydrazine-Hydrazide-Linked Covalent Organic Frameworks for Water Harvesting. *ACS Cent. Sci.* **2022**, *8*, 926–932.

(23) Sun, C.; Zhu, Y.; Shao, P.; Chen, L.; Huang, X.; Zhao, S.; Ma, D.; Jing, X.; Wang, B.; Feng, X. 2D Covalent Organic Framework for Water Harvesting with Fast Kinetics and Low Regeneration Temperature. *Angew. Chem. Int. Ed.* **2023**, *62*, e202217103.

(24) Nguyen, H. L.; Hanikel, N.; Lyle, S. J.; Zhu, C.; Proserpio, D. M.; Yaghi, O. M. A Porous Covalent Organic Framework with Voided Square Grid Topology for Atmospheric Water Harvesting. *J. Am. Chem. Soc.* **2020**, *142*, 2218–2221.

(25) Chen, L.; Han, W.; Yan, X.; Zhang, J.; Jiang, Y.; Gu, Z. A Highly Stable Ortho - Ketoenamine Covalent Organic Framework with Balanced Hydrophilic and Hydrophobic Sites for Atmospheric Water Harvesting. *ChemSusChem* **2022**, *15*, e202201824.

(26) Hanikel, N.; Pei, X.; Ccheda, S.; Lyu, H.; Jeong, W.; Sauer, J.; Gagliardi, L.; Yaghi, O. M. Evolution of Water Structures in Metal-Organic Frameworks for Improved Atmospheric Water Harvesting. *Science* **2021**, *374*, 454–459.

(27) Hanikel, N.; Kurandina, D.; Chheda, S.; Zheng, Z.; Rong, Z.; Neumann, S. E.; Sauer, J.; Siepmann, J. I.; Gagliardi, L.; Yaghi, O. M. MOF Linker Extension Strategy for Enhanced Atmospheric Water Harvesting. *ACS Cent. Sci.* **2023**, *9*, 551–557.

(28) Zheng, Z.; Alawadhi, A. H.; Chheda, S.; Neumann, S. E.; Rampal, N.; Liu, S.; Nguyen, H. L.; Lin, Y.; Rong, Z.; Siepmann, J. I.; Gagliardi, L.; Anandkumar, A.; Borgs, C.; Chayes, J. T.; Yaghi, O. M. Shaping the Water-Harvesting Behavior of Metal–Organic Frameworks Aided by Fine-Tuned GPT Models. *J. Am. Chem. Soc.* **2023**, *145*, 28284–28295.

(29) Alawadhi, A. H.; Chheda, S.; Stroscio, G. D.; Rong, Z.; Kurandina, D.; Nguyen, H. L.; Rampal, N.; Zheng, Z.; Gagliardi, L.; Yaghi, O. M. Harvesting Water from Air with High-Capacity, Stable Furan-Based Metal–Organic Frameworks. *J. Am. Chem. Soc.* **2024**, *146*, 2160–2166.

(30) Lenzen, D.; Zhao, J.; Ernst, S.-J.; Wahiduzzaman, M.; Ken Inge, A.; Fröhlich, D.; Xu, H.; Bart, H.-J.; Janiak, C.; Henninger, S.; Maurin, G.; Zou, X.; Stock, N. A Metal–Organic Framework for Efficient Water-Based Ultra-Low-Temperature-Driven Cooling. *Nat. Commun.* **2019**, *10*, 3025.

(31) Cadiau, A.; Lee, J. S.; Damasceno Borges, D.; Fabry, P.; Devic, T.; Wharmby, M. T.; Martineau, C.; Foucher, D.; Taulelle, F.; Jun, C.-H.; Hwang, Y. K.; Stock, N.; De Lange, M. F.; Kapteijn, F.; Gascon, J.; Maurin, G.; Chang, J.-S.; Serre, C. Design of Hydrophilic Metal Organic Framework Water Adsorbents for Heat Reallocation. *Adv. Mater.* **2015**, *27*, 4775–4780.

(32) Furukawa, H.; Gándara, F.; Zhang, Y.-B.; Jiang, J.; Queen, W. L.; Hudson, M. R.; Yaghi, O. M. Water Adsorption in Porous Metal–Organic Frameworks and Related Materials. *J. Am. Chem. Soc.* **2014**, *136*, 4369–4381.

(33) Frisch, M. J.; Trucks, G. W.; Schlegel, H. B.; Scuseria, G. E.; Robb, M. a.; Cheeseman, J. R.; Scalmani, G.; Barone, V.; Petersson, G. a.; Nakatsuji, H.; Li, X.; Caricato, M.; Marenich, a. V.; Bloino, J.; Janesko, B. G.; Gomperts, R.; Mennucci, B.; Hratchian, H. P.; Ortiz, J. V.; Izmaylov, a. F.; Sonnenberg, J. L.; Williams; Ding, F.; Lipparini, F.; Egidi, F.; Goings, J.; Peng, B.; Petrone, A.; Henderson, T.; Ranasinghe, D.; Zakrzewski, V. G.; Gao, J.; Rega, N.; Zheng, G.; Liang, W.; Hada, M.; Ehara, M.; Toyota, K.; Fukuda, R.; Hasegawa, J.; Ishida, M.; Nakajima, T.; Honda, Y.; Kitao, O.; Nakai, H.; Vreven, T.; Throssell, K.; Montgomery Jr., J. a.; Peralta, J. E.; Ogliaro, F.; Bearpark, M. J.; Heyd, J. J.; Brothers, E. N.; Kudin, K. N.; Staroverov, V. N.; Keith, T. a.; Kobayashi, R.; Normand, J.; Raghavachari, K.; Rendell, a. P.; Burant, J. C.; Iyengar, S. S.; Tomasi, J.; Cossi, M.; Millam, J. M.; Klene, M.; Adamo, C.; Cammi, R.; Ochterski, J. W.; Martin, R. L.; Morokuma, K.; Farkas, O.; Foresman, J. B.; Fox, D. J. G16_C01. 2016, p Gaussian 16, Revision C.01, Gaussian, Inc., Wallin.

(34) Zhao, Y.; Truhlar, D. G. A New Local Density Functional for Main-Group Thermochemistry, Transition Metal Bonding, Thermochemical Kinetics, and Noncovalent Interactions. *J. Chem. Phys.* **2006**, *125*, 194101.

(35) Weigend, F.; Ahlrichs, R. Balanced Basis Sets of Split Valence, Triple Zeta Valence and Quadruple Zeta Valence Quality for H to Rn: Design and Assessment of Accuracy. *Phys. Chem. Chem. Phys.* **2005**, *7*, 3297–3305.

(36) Weigend, F. Accurate Coulomb-Fitting Basis Sets for H to Rn. *Phys. Chem. Chem. Phys.* **2006**, *8*, 1057–1065.
